# Knockout of vascular smooth muscle EGF receptor in a mouse model prevents obesity-induced vascular dysfunction and renal damage in vivo

**DOI:** 10.1007/s00125-020-05187-4

**Published:** 2020-06-17

**Authors:** Christian Stern, Barbara Schreier, Alexander Nolze, Sindy Rabe, Sigrid Mildenberger, Michael Gekle

**Affiliations:** Julius Bernstein Institute of Physiology, Martin Luther University Halle-Wittenberg, Magdeburger Strasse 6, 06112 Halle, Germany

**Keywords:** Diabetes mellitus, EGFR, Epidermal growth factor receptor, Renal damage, Serum response factor, Vascular dysfunction

## Abstract

**Aims/hypothesis:**

Obesity causes type 2 diabetes leading to vascular dysfunction and finally renal end-organ damage. Vascular smooth muscle (VSM) EGF receptor (EGFR) modulates vascular wall homeostasis in part via serum response factor (SRF), a major regulator of VSM differentiation and a sensor for glucose. We investigated the role of VSM-EGFR during obesity-induced renovascular dysfunction, as well as EGFR–hyperglycaemia crosstalk.

**Methods:**

The role of VSM-EGFR during high-fat diet (HFD)-induced type 2 diabetes was investigated in a mouse model with inducible, VSM-specific EGFR-knockout (KO). Various structural and functional variables as well as transcriptome changes, in vivo and ex vivo, were assessed. The impact of hyperglycaemia on EGFR-induced signalling and SRF transcriptional activity and the underlying mechanisms were investigated at the cellular level.

**Results:**

We show that VSM-EGFR mediates obesity/type 2 diabetes-induced vascular dysfunction, remodelling and transcriptome dysregulation preceding renal damage and identify an EGFR–glucose synergism in terms of SRF activation, matrix dysregulation and mitochondrial function. EGFR deletion protects the animals from HFD-induced endothelial dysfunction, creatininaemia and albuminuria. Furthermore, we show that HFD leads to marked changes of the aortic transcriptome in wild-type but not in KO animals, indicative of EGFR-dependent SRF activation, matrix dysregulation and mitochondrial dysfunction, the latter confirmed at the cellular level. Studies at the cellular level revealed that high glucose potentiated EGFR/EGF receptor 2 (ErbB2)-induced stimulation of SRF activity, enhancing the graded signalling responses to EGF, via the EGFR/ErbB2–ROCK–actin–MRTF pathway and promoted mitochondrial dysfunction.

**Conclusions/interpretation:**

VSM-EGFR contributes to HFD-induced vascular and subsequent renal alterations. We propose that a potentiated EGFR/ErbB2–ROCK–MRTF–SRF signalling axis and mitochondrial dysfunction underlie the role of EGFR. This advanced working hypothesis will be investigated in mechanistic depth in future studies. VSM-EGFR may be a therapeutic target in cases of type 2 diabetes-induced renovascular disease.

**Data availability:**

The datasets generated during and/or analysed during the current study are available in: (1) share_it, the data repository of the academic libraries of Saxony-Anhalt (10.25673/32049.2); and (2) in the gene expression omnibus database with the study identity GSE144838 (https://www.ncbi.nlm.nih.gov/geo/query/acc.cgi?acc=GSE144838).

**Figure:**
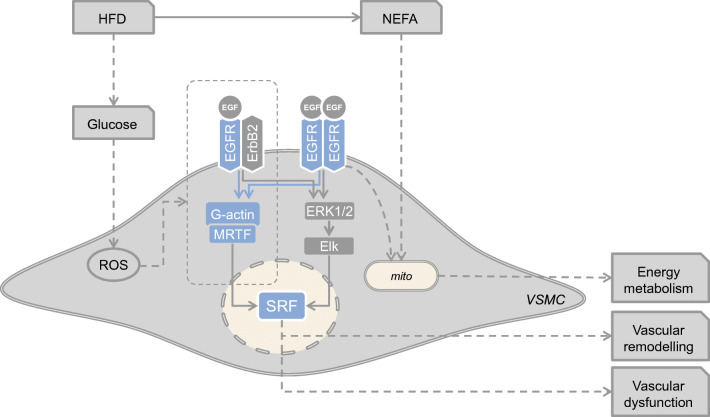
Graphical abstract

**Electronic supplementary material:**

The online version of this article (10.1007/s00125-020-05187-4) contains peer-reviewed but unedited supplementary material, which is available to authorised users.

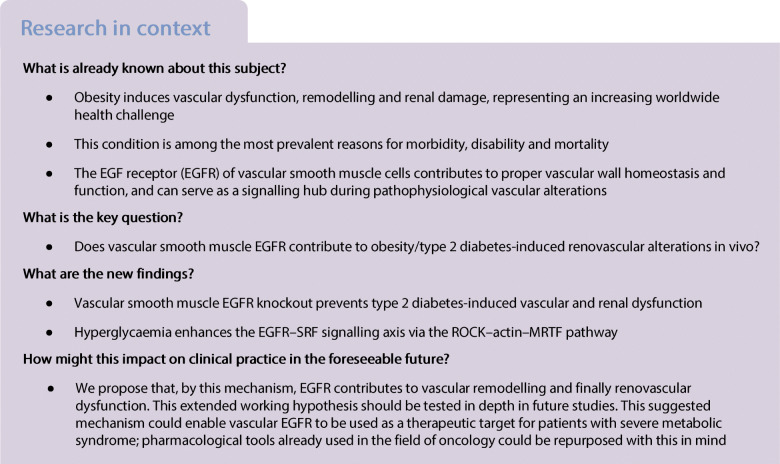



## Introduction

The EGF receptor (EGFR; also named ErbB1) is a member of the ErbB-receptor tyrosine kinase family, which also includes ErbB2 (EGF receptor 2, which is able to form heterodimers with EGFR), ErbB3 and ErbB4. EGFR is activated by EGF or heparin-bound EGF (HB-EGF), modulating cell differentiation, migration and matrix homeostasis [1]. EGFR can also be transactivated, thereby contributing to cardiovascular dysfunction and remodelling [2, 3]. Recently, the relevance of vascular smooth muscle (VSM)-EGFR for structural and functional vascular remodelling, as well as for subsequent complete renal end-organ damage, was shown [4]. Furthermore, pharmacological EGFR inhibition can prevent vascular remodelling [5, 6].

A contribution of VSM-EGFR to obesity- and type 2 diabetes-associated and other vascular alterations [3, 7–11], as well as enhanced vascular EGFR activity during hyperglycaemia with pathophysiological relevance, has been described. In addition, systemic EGFR-kinase inhibitors improved vascular function in diabetic animals [8–14]. There is also some, albeit ambiguous, evidence for an impact of NEFA on EGFR [15–17]. Altogether, these findings raise a question regarding the pathogenetic importance of VSM-EGFR during obesity/type 2 diabetes.

Serum response factor (SRF) is a transcriptional regulator of vascular smooth muscle cells (VSMCs) [18, 19] linking growth factor signalling to pathological transformation into a proliferative phenotype [20, 21]. SRF is at the confluence of multiple signalling pathways via the extracellular signal-regulated kinase (ERK)1/2 or actin–myocardin-related transcription factor (MRTF) pathway [22]. In addition, SRF has been proposed to regulate cellular energy metabolism [23, 24] and to mediate glucose- and lipid-induced gene expression alterations in VSMCs and diabetic nephropathy [25]. Thus, SRF represents a potential integration hub of EGFR and glucose-induced signalling during obesity/type 2 diabetes.

Recently, we established a knockout mouse model with inducible deletion of the EGFR in VSMCs [26] and showed the physiological and pathophysiological importance of VSM-EGFR in vivo [4, 26]. The present study investigates the role of VSM-EGFR during high-fat diet-induced obesity/type 2 diabetes regarding vascular remodelling, gene expression and renal damage using this genetic model.

## Methods

All mouse experiments were approved by the local government (Landesverwaltungsamt Sachsen-Anhalt, Germany, Az.: 505.6.3-42502-2-1389 MLU_G; Veterinäramt Stadt Halle, Germany; Bescheid T16/2019) and conducted in accordance with the National Institutes of Health Guide for the Care and Use of Laboratory Animals, the ARRIVE guidelines and with consideration of the 3R-principle.

### Animals

Mice were kept at constant temperature of 22 ± 2°C, relative humidity of 30–60%, under a 12/12 h light–dark cycle with ad libitum access to water and standard chow. Recently, we generated and described an inducible knockout (KO) for EGFR in VSMCs via the Cre/loxP system by mating *Egfr*^*flox/flox*^ C57BL/6 mice (originally provided by M. Sibilia, Institut für Krebsforschung - MedUni Wien, Vienna, Austria) with *Smmhc-Cre*^*+/*^*-* C57BL/6N mice (B6.FVB-Tg(Myh11-cre/ERT2)1Soff/J, originally provided by S. Offermanns, Max Planck Institute, Bad Nauheim, Germany; *Smmhc* is also known as *Myh11*) [4, 26]. In the *iEgfr*^Δ/ΔVSMC^ littermates Cre-recombinase is located on the Y chromosome, expressed under control of the *Smmhc*-promoter and can be activated in VSMCs by intraperitoneal injection of tamoxifen (1 mg of 50 μl Miglyol 812/mouse per day for 5 consecutive days). As controls, wild-type (WT) mice without LoxP-sites but carrying Cre-recombinase under the control of the *Smmhc* promotor and treated with tamoxifen were employed. All experiments were performed in male animals. High-fat diet (HFD) was started 7 days after tamoxifen application in mice, at 6 weeks of age. Genotyping was performed on tail biopsies by PCR as previously described [2]. Breeding of the animals and assignment to experimental groups was performed randomly by place holder numbers before information regarding the animals was obtained. During further experimentation the genotype of the animals was blinded by pseudonymisation (assignment of numbers). Owing to the differences in weight gain the type of diet could not be blinded.

### Cell culture

HK-2 (a human proximal tubule epithelial cell line, CRL-2190) and HEK293 (human embryonic kidney cell line, CRL-3216) were obtained for this project from ATCC, USA. Both cell types were cultivated in DMEM/Ham’s F-12 medium (FG 4815, Biochrom, Berlin, Germany), supplemented with 10% FCS. Medium was changed to DMEM without FCS prior to addition of stimuli. A7r5 cells (also obtained for this project from ATCC, CRL-1444) were cultivated in DMEM. The cells were authenticated by the supplier (ATCC) and kept in separate cultures after arrival to avoid cross-contamination. Verification was continued by cell morphology monitoring throughout the project. The mycoplasma contamination status was negative as determined by routine mycoplasma detection PCR.

Primary culture of murine VSMCs was obtained from KO and WT mice, as described by Ray et al [27]. Thoracic and abdominal aorta was excised in 0.9% sterile NaCl, cleared of blood as well as surrounding tissue and rinsed several times. Subsequently the aorta was transferred to DMEM (+10% FCS), reduced mechanically to small pieces and digested in collagenase 2 (in DMEM, 4–6 h at 37°C, 5% CO_2_). Thereafter cells were gently dispersed and rinsed with medium. After 5 min centrifugation at 300 *g* at room temperature the cell pellet was resuspended in 5 ml fresh medium and centrifuged a second time. Finally, the cells were seeded in plastic dishes in DMEM containing 10% serum and incubated for 5 days at 37°C, 5% CO_2_.

### Measurement of aortic ring force

Aortic rings were equilibrated in modified aerated Krebs–Ringer solution (20% O_2_, 5% CO_2_) at 37°C for 30 min. At the beginning and the end of the equilibration the physiological salt solution was changed once, followed by the application of a strain resulting in a force of 12 mN [4, 26]. This strain resulted in a similar change in vessel circumference (delta length [dL]) and similar effective pressure values in both genotypes and was applied for 10 min prior to the first substance application. Wall stress and effective pressure were calculated as described by Mulvany and Halpern [28]. After each measurement the chambers were flushed five times with Krebs–Ringer solution, achieving an approximately 100,000-fold dilution of the substance, before a new reagent was tested. This did not apply for the relaxants carbamoylcholine chloride (carbachol) and *S*-Nitroso-*N*-acetyl-dl-penicillamine (SNAP). These substances were given at the point of stable force development of the previously administered vasoconstrictor. Vasoconstriction was induced by phenylephrine, U46619 (both for pharmacomechanical coupling) or KCl (electromechanical coupling).

### Pressurised mesenteric arteries

Pressure myography was performed using a DMT system (Aarhus, Denmark) [4]. After mice were killed, the mesenteric bed was removed and transferred to cold (4°C), gassed (20% O_2_, 5% CO_2_) physiological salt solution. Mesenteric (third or fourth order) arteries were mounted on glass cannulas to allow perfusion at physiological pressures (inlet pressure 60 mmHg, outlet pressure 45 mmHg). Vessels were superfused continuously with Krebs–Henseleit solution (20% O_2_, 5% CO_2_; pH, 7.4; 37°C) composed of (mmol/l): 119 NaCl, 4.7 KCl, 25 NaHCO_3_, 1.2 KH_2_PO_4_, 1.6 CaCl_2_, 1.2 MgSO_4_, 0.03 EDTA and 11.1 glucose. The wake-up procedure was carried out according to the manufacturer’s instructions by stepwise pressurising to 20, 40, 60, 80 or 100 mmHg using servo control system. The diameter of the vessels was measured with a video microscope (Zeiss Axiovert, Oberkochen, Germany) and a data acquisition and analysis system (Danish Myo Technology A/S, DK-8382 Hinnerup, Denmark). After 45–60 min equilibration a 60 mmol/l KCl challenge was performed before any other interventions.

### Harvesting of organs

Mice were killed by cervical dislocation, and livers, kidneys, lungs, hearts and aortas were excised, carefully freed from adjacent tissue, and partially weighed. Tibia length was measured for normalisation of organ weights. Parts of the tissues were immediately snap frozen in liquid nitrogen while parts were fixed in 5% paraformaldehyde solution. Tissues were dehydrated in increasing concentrations of methanol or isopropanol. After embedding in paraffin, 4 μm sections were cut [4].

### Histomorphometric analysis of aorta, heart, kidney and cardiomyocytes

Morphometric analysis was performed as described before [2, 4] in a blinded way (pseudonymisation by number assignment). For media-to-lumen ratio, media thickness was measured at ten different locations within the vessel wall and divided by the internal circumference of the aorta as described by Liang et al [29]. Fibrosis was analysed by staining with Sirius red followed by quantitative microscopic determination of the relative fibrotic area. Perivascular fibrosis was normalised to the respective vascular cross-sectional area [30]. Interstitial fibrosis was normalised to the area of tissue under investigation [31, 32]. Sections were investigated in a double-blinded manner. Glomerular damage was assessed as Bowman and glomerular area, glomerular cellularity (nuclei per glomerulus) and glomerulosclerosis in kidney sections stained with periodic acid–Schiff’s (PAS) reagent or H&E. At least 30 glomeruli per kidney were evaluated, and the values are given as the mean score per animal. The degree of glomerulosclerosis was determined using a semiquantitative scoring method [33]. Glomeruli were selected randomly and scored as follows: grade 0, normal; grade 1, sclerotic area 25% of total glomerular area; grade 2, sclerotic area 25–50%; grade 3, sclerotic area 50–75%; grade 4, sclerotic area 75–100%. The degree of proximal tubular vacuolisation was also determined using a semiquantitative scoring method: 35 images per kidney cortex (PAS staining) were selected randomly and scored as follows: grade 0, <10% vacuolisation; grade 1, 10–20%; grade 2, 20–30%; grade 3, 30–40%; grade 4, 40–50% and grade 5, >50%.

### Next generation sequencing and gene enrichment analysis

Total RNA was isolated as described [34, 35]. Sequencing of aortic samples was performed with an Illumina HiSeq2000 at the Core Unit DNA Technologies of the Medical Faculty, University of Leipzig (Leipzig, Germany). Libraries were prepared with indexed adapters, and clusters were generated on the cluster flow cells. cDNA fragments were hybridised to the lawn of complementary primers followed by bridge amplification. Paired-end sequencing by synthesis (SBS) was performed via reversible terminator-based method. A quality check was performed on raw data (fastQC, v0.11.3, https://www.bioinformatics.babraham.ac.uk/projects/fastqc/) before adapter clipping and quality trimming (cutadapt, v1.8.1) [36] and a further quality check (fastQC) followed by alignment against the murine genome (assembly GRCm38/mm10). Initial mapping was done with Bowtie2 (2.2.5) [37] followed by Tophat2 (2.0.14) [38] using Bowtie to align spliced reads. Finally counting was done with featureCounts (1.4.6) [39] and genes were annotated with BiomaRt v93 (R package v2.36.1) [40]. Normalisation and differential expression analysis were performed using R package EdgeR (3.20.8) [41] from Bioconductor (https://www.bioconductor.org/). For the analysis with EdgeR, the counts were normalised using the trimmed mean of M values (TMM) method. A false discovery rate (FDR) of 0.001 was used to determine if genes were significantly regulated. Sequencing of kidney samples was performed on an Illumina System by Novogene (Cambridge, UK). Raw data quality check, adapter clipping and quality trimming were performed externally by Novogene, followed by the previously described analysis pipeline. The raw data are available at https://www.ncbi.nlm.nih.gov/geo/query/acc.cgi?acc=GSE144838.

mRNA enrichment analysis was performed using g:Profiler (http://biit.cs.ut.ee/gprofiler/ [42]) and GOrilla (http://cbl-gorilla.cs.technion.ac.il/ [43]). Transcription factor binding site (TFBS) enrichment within the promotor regions (–950 to +50 and –450 to +50) of differentially expressed mRNAs was evaluated by Pscan (http://159.149.160.88/pscan/ [44]). Ingenuity Pathway Analysis (IPA) software (Qiagen, Hilden, Germany) was used for functional analysis (canonical pathways, upstream regulator and downstream effects analyses; these features are not included in g:Profiler or GOrilla) on the lists of regulated genes (results of the differential expression analyses). Their Ensembl identifiers were mapped to networks available in the software database. For the canonical pathway analysis, enriched pathways were ranked according to how relevant they were for the genes provided as input. Multiple testing was performed using the Benjamini–Hochberg (B–H) procedure. Analyses were corrected for multiple testing as described for the corresponding tools.

### Reporter gene analysis

Reporters for *Srf* (sequence GGATGTCCATATTAGGA), *Egr1* (sequence CGCCCCCGCG), *Ap1* (also known as *Jun*; sequence TGAGTCAG) or *Nfkb* (sequence GGGACTTTCC) transcription factors were purchased from Qiagen. We used the CignalTM System (www.qiagen.com/lu/products/discovery-and-translational-research/functional-and-cell-analysis/gene-reporter-assays/cignal-reporter-assay-kits/#productdetails) with Monster-green fluorescent protein (MGFP, improved synthetic version of the green fluorescent protein with enhanced fluorescence and reduced cytotoxicity) as reporter. The respective transfection control was red fluorescent protein (RFP) under the control of a constitutive cytomegalovirus (CMV) promoter. After transfection with Polyfect (Qiagen), cells were incubated as described in the figure legends and reporter activity was determined as recommended by the manufacturer by digital fluorescence microscopy (Cytation 3, BioTek, Bad Friedrichshall, Germany or the PerkinElmer [USA] Operetta CLS high content screening system). To determine the cellular responses, first transfected cells were identified according to their red fluorescence and their number, and mean fluorescence intensity, area, circularity and fluorescence integral were determined. Second, the mean green fluorescence intensity of the red cells as well as the green fluorescence integral of red cells was determined. Finally, red cells that were also green were identified and their number, mean fluorescence intensity, area, circularity and fluorescence integral was determined. SRF activity is expressed either as the ratio of fluorescence (green) derived from the serum response element (SRE)-GFP reporter over transfection control RFP fluorescence, when the comparison over several passages was relevant, or as per cent of the control in the same experiment after correction for RFP fluorescence.

### Quantitative RT-PCR

Total RNA for RT-qPCR was isolated using InviTrap spin tissue RNA mini kit (Invitek Molecular, Germany) following manufacturer’s instructions. DNA contamination was removed (DNAse I, New England Biolabs, USA) and reverse transcription was performed using random primers and SuperScript II reverse transcriptase (Invitrogen, Life Technologies, USA) according to the manufacturer’s instructions. A 1 μL aliquot of the obtained cDNA was used in RT-qPCR (AriaMx Real-Time PCR System, Agilent Technologies, Germany). Primer sequences are available in electronic supplementary material (ESM) Table 1. qPCR efficiency was >90%. The relative mRNA expression of the genes of interest was calculated according to the ${2}^{-{\Delta \Delta \mathrm{C}}_{\mathrm{t}}}$ method, using the 18S RNA signal for normalisation. Each sample was analysed in triplicate. Values are expressed as mean difference between WT and KO ± SEM.

### Immunoblotting

For protein expression level determination, cells were lysed with CST lysis buffer (20 mmol/l Tris, pH 7.5 [Illinois Tools Works companies], 150 mmol/l NaCl [Applichem], 1% Triton X-100 [Sigma-Aldrich], 1 mmol/l EDTA [Merck], 1 mmol/l EGTA [Sigma-Aldrich], 184 mg/l Na-orthovanadate [Sigma-Aldrich], 2.5 mmol/l Na-pyrophosphate [Sigma-Aldrich], 1 mmol/l β-glycerolphosphate [Sigma-Aldrich]), centrifuged at 13,000 *g* for 10 min and protein amount was determined with Bradford assay. Equal amounts of the proteins were denaturated with 6x Laemmli buffer (0.5 mol/l Tris pH 6.8 [Roth], 10% SDS [Roth], 10% glycerol [Sigma-Aldrich]) at 95°C for 5–10 min. Proteins were separated by 10% SDS-PAGE and transferred onto a nitrocellulose membrane. After blocking with 5% nonfat dry milk powder in Tris-buffered saline with Tween20 (TBS-Tween) (20 mmol/l Tris base, pH 7.4 [Illinois Tools Works companies], 150 mmol/l NaCl [Applichem], 0.05% Tween-20 [Sigma-Aldrich]) membranes were incubated with first antibody (see below) diluted in 5% BSA in TBS-Tween overnight. Horseradish peroxidase (HRP)-coupled secondary antibodies, 1:1000 in 5% skimmed (nonfat) dry milk powder in TBS-Tween were used. After removal of non-bonded secondary antibody, three washing steps in TBS-Tween were performed. Finally Clarity Western ECL Substrate (BioRad, Munich, Germany) was added and the peroxidase activity-based light emission was recorded by an imaging system (Image Quant LAS4000, GE Healthcare, UK). All antibodies were purchased from Cell Signaling Technologies, Frankfurt, Germany (phosphoERK1/2 #9101, ERK1/2 #9102, EGFR #2232, EGFR XP #4267, ErbB2 #4290, SRF #5147, β-actin #4967, early growth response (EGR)1 #4153, GAPDH #2118, anti-rabbit-HRP #7074). Densitometry analysis was performed with Quantity One software from BioRad (Feldkirchen, Germany) and the relative expression values calculated using β-actin or GAPDH expression as housekeepers.

### Determination of albumin, creatinine, glucose and lactate

Glucose consumption and lactate production were assessed using commercially available kits (Glucose [HK] assay kit, Sigma, Darmstadt, Germany and Lactate Reagent, Trinity Biotech, via Menarini Diagnostics, Berlin, Germany) according to the manufacturer’s instructions [34]. Albumin was determined by ELISA (Bethyl Laboratories, Montgomery, TX, USA). Creatinine was determined enzymatically by the creatinase method (Diazyme, Dresden, Germany).

### Determination of mitochondrial potential, reactive oxygen species and oxygen consumption rate

Assessment of alterations of mitochondrial potential was performed using a mitochondrial membrane potential assay kit (Cayman Chemical, USA) that employs mitochondrial potential-dependent dye JC-10. After incubation of the cells as indicated, 50 μl of JC-10 dye was added per 1 ml media and the plate was immediately placed into a digital fluorescence microscope (Cytation 3, BioTek, Bad Friedrichshall, Germany) heated to 37°C. Fluorescence of JC-10 (excitation 405 nm; emission 529 nm for JC10-aggregates indicating healthy mitochondria and 590 nm for JC-1 monomers indicating reduced mitochondrial potential) was determined after 30 min. The surrogate parameter for mitochondrial potential is the 529/590 nm ratio, with higher values indicating higher (more negative) mitochondrial membrane potential. Oxygen consumption rate (OCR) was determined by Seahorse technology using the manufacturer’s assay kit (Seahorse XFe96 Analyzer, Agilent Technologies, Waldbronn, Germany). OCR was normalised for cell density by nuclear staining with Hoechst 33342 (2 μmol/l). Formation of reactive oxygen species (ROS) was estimated from the changes in MitoSox fluorescence (Life Technologies, Darmstadt, Germany; excitation 510 nm; emission 580 nm) normalised to the cellular protein amount [45].

### Chemicals

AG1478 (inhibitor of EGFR), AG879 (inhibitor of ErbB2) and tiron (scavenger of ROS) were obtained from Merck (Germany), U0126 (inhibitor of ERK1/2 activation) and latrunculin B (sequesters G-actin and prevents F-actin assembly) from Cayman Chemicals (USA), Y27632 (inhibitor of Rho kinase) from Tocris Bioscience (USA).

### Statistics

Data are presented as mean ± SEM. A two-way ANOVA: standard-fat diet (SFD) vs HFD and WT vs KO was performed. Followed by post hoc testing, Student’s *t* test or Mann–Whitney rank sum test were used as applicable according to pre-test data analysis by SigmaPlot 12.5. A *p* value <0.05 was considered significant. *n* = number of animals or cell culture dishes. Biometrical planning was performed under consideration of the 3R-principle with α = 0.05 and β = 0.8, resulting in sample sizes between 8 and 11. For cell culture experiments, at least three different passages were used. Data from all experiments that proceeded technically according to plan were included into the analyses.

## Results

### Animal studies

#### Systemic variables

**Fig. 1:**
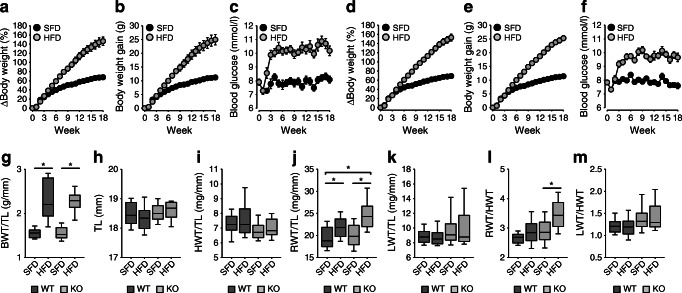
Influence of HFD on body weight, blood glucose and organ weight. (**a**–**f**) Change in body weight, body weight gain and blood glucose levels in WT animals (**a**–**c**) and KO animals (**d**–**f**). Weeks are counted from the start of feeding HFD. (**g**–**m**) Organ weight values obtained at week 18. Number of animals: WT-SFD *n* = 23; WT-HFD *n* = 24; KO-SFD *n* = 38; KO-HFD *n* = 40. *p < 0.05 vs respective control, unless indicated differently. BWT, body weight; HWT, heart weight; LWT, lung weight; RWT, renal weight; TL, tibia length

Vascular EGFR expression is reduced substantially in VSM-*Egfr* KO mice [4, 26]. KO mice showed no difference in body weight, tibia length or blood glucose compared with WT littermates (Fig. 1). HFD led to similar increases in body weight and blood glucose in both genotypes (Fig. 1g,h) but induced a larger increase of renal weight in KO mice (Fig. 1i–m).

#### Functional vascular parameters

**Fig. 2:**
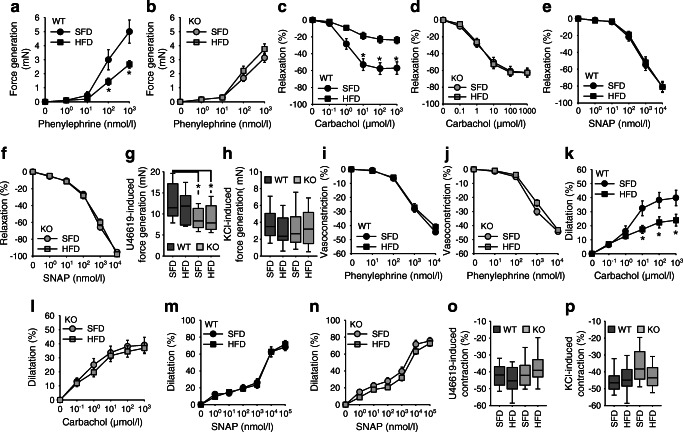
(**a**–**h**) Isometric force generation and relaxation of aortic rings in WT and KO mice fed an HFD or SFD, as shown. Number of animals: WT-SFD *n* = 8; WT-HFD *n* = 8; KO-SFD *n* = 12; KO-HFD *n* = 14. (**i**–**p**) Pressure myography in mesenteric arteries of WT and KO mice fed an HFD or SFD, as shown. *n* = 8 animals for each experimental group. All values were obtained at week 18. (**a**–**f**, **i**–**n**) *x*-axes are plotted on a log scale. *p* < 0.05 vs SFD, unless indicated differently

Isometric force measurements in aortic rings revealed a smaller response to the α1-adrenergic agonist phenylephrine in KO animals following SFD feeding (Fig. 2a,b). The same was true for the thromboxane analogue U46619 (Fig. 2g) but not for depolarisation by KCl (Fig. 2h). HFD reduced phenylephrine-induced force generation of WT but not of KO rings (Fig. 2a,b). Endothelium-dependent relaxation induced by carbachol was reduced by HFD in aortic rings from WT, yet not from KO mice (Fig. 2c,d). There were no differences in endothelium-independent relaxation induced by SNAP (Fig. 2e,f).

Pressure myography in mesenteric arteries showed no effect of diet or genotype on the response to phenylephrine, U46619 or KCl (Fig. 2i,j,o,p). HFD induced endothelial dysfunction in arteries from WT but not from KO mice (Fig. 2k,l), whereas relaxation induced by SNAP was not affected (Fig. 2m,n).

#### Aortic gene expression analysis

KO led to differential expression of 89 mRNAs following SFD feeding that showed only few gene ontology (GO) terms and no TFBS enrichment (the aorta raw data table can be found in our data repository; 10.25673/32049.2). We observed fourfold greater enrichment (adjusted *p* value <0.05) for: (1) transmembrane receptor protein tyrosine kinase activity (GO:0004714); (2) enzyme linked receptor protein signalling pathway (GO:0007167); (3) mitogen-activated protein kinase (MAPK) cascade (GO:0000165); (4) regulation of MAPK cascade (GO:0043408); and (5) signal transduction by protein phosphorylation (GO:0023014), all of which reflected *Egfr* KO. Enhanced expression of *Rgs5*, relevant for contraction regulation [46–48], in KO animals (aorta raw data table; 10.25673/32049.2) might explain altered responses to phenylephrine and U46619 (Fig. 2a,b,g). However, at present we can only propose this hypothesis because we did not perform further experiments to test the impact of *Rgs5* upregulation.

**Fig. 3:**
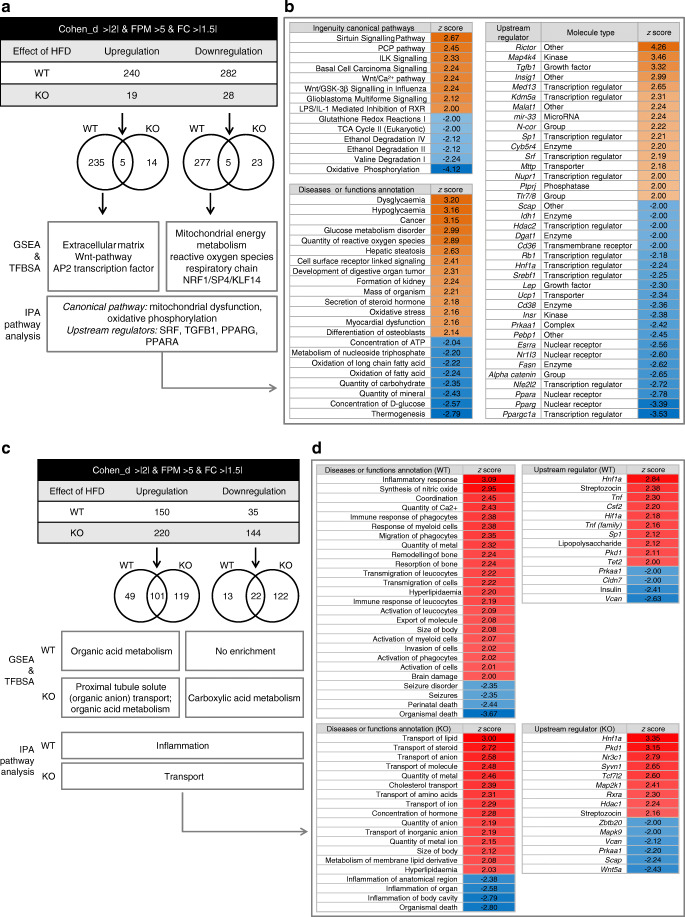
(**a**) Vascular gene expression analysis using mouse aortae. Overview of quantitative HFD effects in the two genotypes using the depicted constraints. Summary of qualitative effects according to GSEA, TFBSA analysis and IPA; *n* = 4 animals for each group. (**b**) IPA pathway analysis for up- and downregulated genes in WT animals. IPA analysis for regulated genes in KO animals yielded no significant results; *n* = 4 animals for each group. (**c**) Unbiased renal gene expression analysis. Overview of the quantitative HFD effects in the two genotypes using the depicted thresholds. Summary of the qualitative effects according to GSEA, TFBSA and IPA; *n* = 4 animals for each group. (**d**) IPA ‘Diseases or functions annotation and upstream regulator’ analysis for up- and downregulated genes in WT and KO animals; *n* = 4 animals for each group. AP2, activator protein 2; FC, fold change; FPM, fragments per million; KLF14, Krüppel-like factor 14; NRF1, nuclear respiratory factor 1; PPAR, peroxisome proliferator-activated receptor; TGFB1, transforming growth factor β1; *Alpha catenin*, also known as *Ctnna1*

HFD induced major changes in mRNA expression in WT aortae (Fig. 3a), with 240 mRNAs upregulated and 282 mRNAs downregulated, including a slight upregulation of *Egfr* and *ErbB2* in WT but not in KO mice (ESM Fig. 1). In KO aortae the number of mRNAs affected by HFD was only ~10% compared with WT. Results of gene set enrichment analysis (GSEA) and TFBS analysis (TFBSA) are presented in Fig. 3a, ESM Fig. 2 and in our online data repository (Table 1A, B; 10.25673/32049.2).

GSEA of downregulated mRNA showed a strong impact on mitochondrial function/oxidative phosphorylation, fatty acid metabolism and ROS generation in WT but not in KO animals (Fig. 3a; online Table 2A, B; 10.25673/32049.2).

IPA pathway analysis confirmed the impact of HFD in WT animals on Wnt signalling, mitochondrial function, ROS generation, and fatty acid and glucose metabolism (Fig. 3b). SRF was identified as a functional upstream regulator, without major differences in SRF mRNA expression (ESM Fig. 1), in WT mice only.

#### EGFR modulates glucose utilisation

**Fig. 4:**
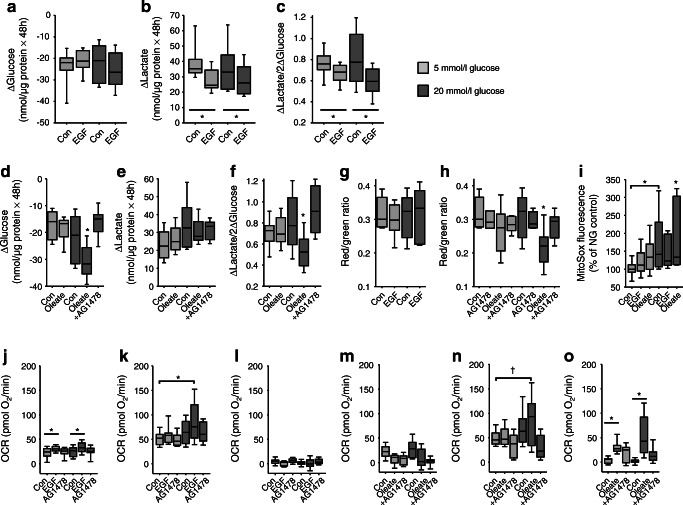
(**a**–**i**) Glucose metabolism (**a**–**f**), mitochondrial potential changes (**g**–**h**) (determined by the red/green fluorescence ratio of the dye JC-10), and estimation of mitochondrial ROS formation (**i**) in VSM A7r5 cells. (**j**–**o**) OCR of A7r5 cells determined by Seahorse technology. OCR was normalised for cell density by nuclear staining with Hoechst 33342 (2 μmol/l). (**j, m**) ATP-linked mitochondrial respiration. (**k, n**) Maximum mitochondrial respiration. (**l, o**) Proton leak. All values were obtained after 48 h incubation.*p < 0.05 vs respective control, unless indicated differently; ^†^*p* = 0.066. Oleate, 100 μmol/l in the presence of fatty acid free BSA; EGF, 10 μg/l; AG1478, 100 nmol/l. Numbers for (**a**–**c**): 5 mmol/l glucose *n* = 28; 20 mmol/l glucose *n* = 16; (**d**–**f**) *n* = 16; (**g**–**h**) *n* = 10; (**i**) *n* = 12; (**j**–**l**) *n* = 18; (**m**–**o**) *n* = 12. ‘+AG1478’ indicates addition of AG1478 in the presence of oleate. Con, control; NG, normal glucose (5 mmol/l)

EGF did not affect glucose consumption but reduced lactate formation and therefore the Δlactate/2Δglucose-ratio in VSMCs A7r5 cells under normal and high glucose conditions (Fig. 4a–c). Addition of the NEFA oleate (Fig. 4d–f) reduced glucose consumption and altered the Δlactate/2Δglucose ratio under high glucose conditions only, an effect prevented by EGFR inhibition with AG1478. Mitochondrial potential was not affected by EGF or glucose (Fig. 4g). Oleate led to an AG1478-sensitive reduction of mitochondrial potential under high glucose conditions (Fig. 4h). High glucose and oleate also increased the rate of mitochondrial ROS formation (Fig. 4i). OCR (Fig. 4j,k,m,n) was stimulated slightly by EGF during ATP-linked mitochondrial respiration and maximum mitochondrial respiration. The negative effect of oleate on mitochondrial function was confirmed by an enhanced proton leak (Fig. 4l,o) and prevented by EGFR inhibition. Finally, maximum mitochondrial OCR of VSMCs in primary culture from KO animals was lower than in WT cells (ESM Fig. 3), confirming the impact of EGFR.

#### Cardiac parameters

The expression of selected cardiac genes was not affected (ESM Fig. 4).

#### Renal parameters

**Fig. 5:**
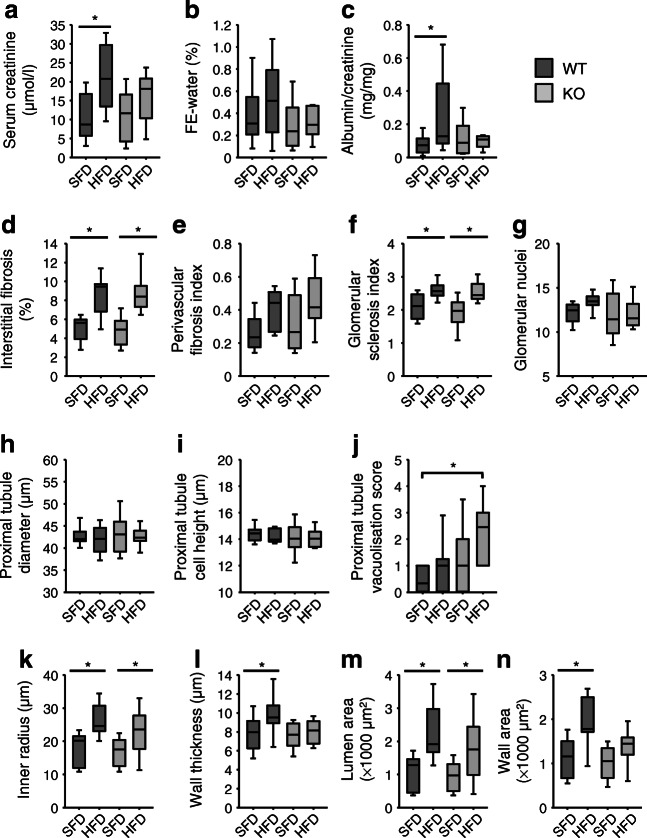
(**a**–**c**) Impact of HFD feeding on functional renal parameters. (**d**–**g**) Impact of HFD on renal interstitial and glomerular parameters. (**h**–**j**) Impact of HFD on proximal tubule parameters. (**k**–**n**) Impact of HFD on renal arteriolar parameters. Number of animals: *n* = 10 for each experimental group. *p < 0.05 vs respective control, unless indicated differently. FE, fractional excretion

HFD led to increased serum creatinine and albuminuria in WT mice only (Fig. 5a–c). The HFD-induced increase in renal weight in KO animals was accompanied by moderate histological changes, including interstitial fibrosis and glomerular sclerosis (Fig. 5d–g) in both genotypes. In contrast, HFD led to increased vacuolisation of proximal tubuli predominantly in KO animals (Fig. 5h–j). Proximal tubule hypertrophy (Fig. 5h,i) or an increase in glomerular size (data not shown) were not observed, but dilated arterioles with thickened walls were observed in WT animals (Fig. 5k–n). Wall thickening was absent in KO animals (Fig. 5l,n), indicative of less remodelling and thereby better perfusion, which explains preserved glomerular filtration.

Expression of a priori selected genes showed only marginal differences (ESM Fig. 5). Consequently, we performed mRNA-sequencing of renal tissue samples.

#### Renal gene expression analysis

In KO kidneys 73 mRNAs were differently expressed following SFD feeding without significant enrichment (aorta raw data table; 10.25673/32049.2). HFD induced differential expression of 185 genes in WT and 364 genes in KO kidneys (Fig. 3c). A substantial fraction of mRNAs was affected only in one of the two genotypes. GSEA and TFBSA results are shown in Fig. 3c and online (Tables 3A, B and 4A, B; 10.25673/32049.2).

IPA analysis confirmed the impact of HFD in KO on transport (Fig. 3d) with positive *z* scores, indicating enhanced transport activity. In WT but not in KO animals, HFD induced an inflammatory response (Fig. 3d), as expected from the literature.

The upstream regulator with a highly significant *p* value of overlap in both genotypes was *Hnf1a* (Fig. 3d), a transcription factor known to regulate transport processes and glucose as well as fatty acid metabolism [49]. In contrast, *Tnf*α and *Csf2* are predicted upstream regulators in WT animals only (Fig. 3d), consistent with an inflammatory response.

### Cell signalling

#### Glucose enhances EGF-induced transcriptional activity of SRF

**Fig. 6:**
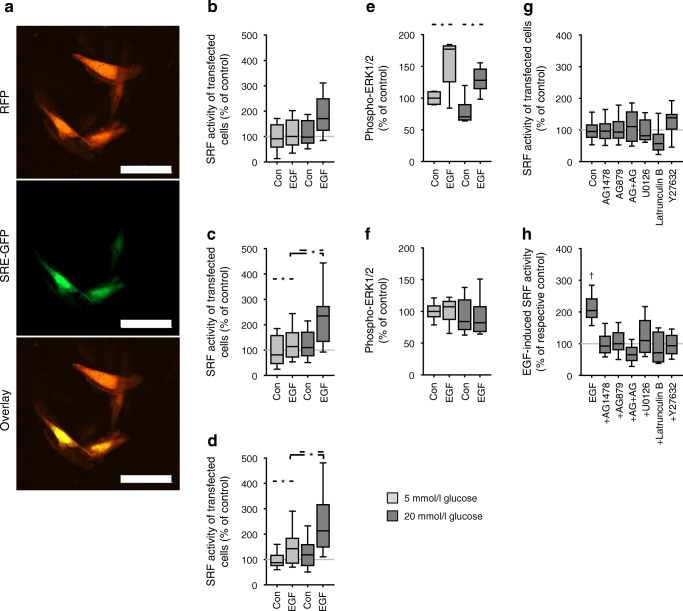
(**a**) Representative fluorescence images of A7r5 cells used for reporter gene analysis. RFP shows the cells successfully transfected that are the basis for the analysis. GFP shows the cells with active SRF (all of which show also red fluorescence). The overlay shows that not all transfected cells have active SRF. Furthermore, the micrographs show that A7r5 cells display a VSMC-typical morphology. Scale bars, 100 μm. Representative images of 30 experiments. (**b–d**) Stimulation of SRF activity in A7r5 cells by EGF (10 μg/l) during 6 h (**b**), 24 h (**c**) and 48 h (**d**) under normal and high glucose conditions determined by reporter gene analysis. High glucose per se exerted no effect. However, high glucose led to a significantly larger EGF effect, i.e. glucose potentiated the EGF action. (**e–f**) ERK1/2 phosphorylation induced by 10 μg/l EGF in A7r5 cells after 24 h (**e**) and 48 h (**f**). (**g, h**) Inhibitor sensitivity of SRF activity in A7r5 cells determined by reporter gene analysis under control conditions without stimulation (**g**) and in the presence of 10 μg/l EGF (**h**). U0126, 1 μmol/l; latrunculin B, 100 nmol/l; AG1478, 100 nmol/l; AG879, 100 nmol/l; Y27632, 10 μmol/l. Con, medium without serum. *p < 0.05 vs respective control, unless indicated otherwise ^†^*p* < 0.05 versus respective inhibitor control (**g**). Numbers for figures (**b–d**) *n* = 30; (**e**) *n* = 6; (**f**) *n* = 8; (**g–h**) *n* = 18

IPA implicated HFD-induced activation of SRF in WT vasculature (Fig. 3b) and concomitant inhibition of PPARγ, known to be regulated negatively by SRF [25]. Enhancing glucose concentration potentiated the EGF effect on SRF transcriptional activity in VSMC (A7r5) cells (Fig. 6b–d) in contrast with NEFA (ESM Fig. 6). Oleate, high glucose or oleate + high glucose did not exerted any effect (ESM Fig. 6).

The effect of glucose is not exclusively attributable to enhanced ERK1/2 phosphorylation, because this was not affected by glucose (Fig. 6e,f and ESM Fig. 7a–c) in A7r5 or primary VSMC cells and SRF activation by EGF was only partially sensitive to inhibition of ERK1/2 activation by U0126 (Fig. 6h, there was still a residual effect of EGF in the presence of U0126, albeit not statistically significant). By contrast, EGF-induced SRF activation was highly sensitive to latrunculin B, an inhibitor of the actin-MRTF pathway (Fig. 6h). Furthermore, EGF-induced SRF activation was sensitive to the ErbB2 inhibitor AG879 (in addition to the EGFR inhibitor AG1478) and we observed an EGF-induced downregulation of ErbB2 in A7r5 cells (Fig. 6h and ESM Fig. 7a,b). In addition, high glucose led to a slight but significant increase in ErbB2 expression (ESM Fig. 7b), indicating the involvement of EGFR–ErbB2 heterodimers. Because inhibition of the Rho/Rho-associated protein kinase (ROCK) pathway by Y27632 (Fig. 6h) prevented EGF-induced SRF activation, we suggest that the EGFR/ErbB2–Rho–ROCK–actin–MRTF pathway is essential.

**Fig. 7:**
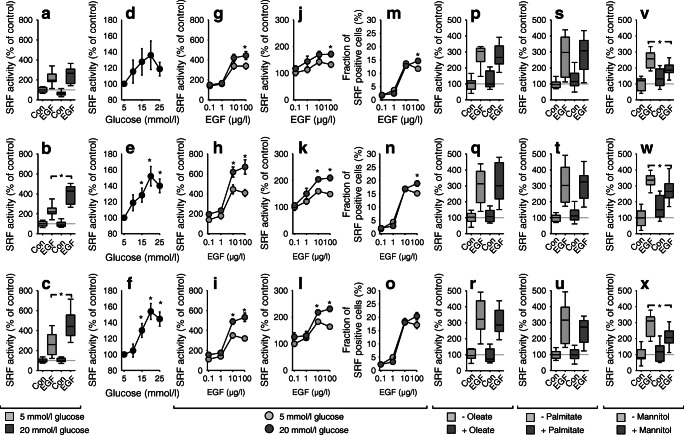
(**a–c**) Stimulation of SRF activity of transfected cells in HK-2 cells by EGF (10 μg/l) and EGF + high glucose after 6 h (**a**), 24 h (**b**) and 48 h (**c**). (**d–f**) In the presence of 10 μg/l EGF, glucose enhances SRF activity of transfected cells in a concentration-dependent manner after 24 h (**e**) and 48 h (**f**). (**g**–**o**) Dose–response curves of EGF-induced SRF activity in HK-2 cells and its alteration by high glucose conditions after 6 h (**g**, **j**, **m**), 24 h (**h**, **k**, **n**) and 48 h (**i**, **l**, **o**). (**g**–**i**) show SRF activity of transfected cells (=total effect); (**j**–**l**) show SRF activity of SRF-positive cells (=analogue effect). Glucose enhances SRF activity mainly by an analogue effect, i.e. enhanced activity in cells already activated by EGF (**j**, **k**, **l**). (**p–x**) 100 μmol/l oleate (**p**–**r**), 100 μmol/l palmitate (**s**–**u**) or 20 mmol/l mannitol (**v**–**x**) exert no stimulatory effect on SRF activity after 6 h (**p**, **s**, **v**), 24 h (**q**, **t**, **w**) and 48 h (**r**, **u**, **x**). *p < 0.05 vs respective control or 5 mmol/l glucose, unless indicated otherwise. Numbers for figures (**a–c**) *n* = 24; (**d–o**) *n* = 24; (**p–r**) *n* = 12; (**s–u**) *n* = 18; (**v–x**) *n* = 12. *x*-axes in figures **g–o** are plotted on a log scale

To test whether the effects are cell specific, we repeated the experiments in HK-2 cells (human renal cells) where glucose also enhanced EGF-induced SRF activity (Fig. 7a–c) starting at ~10 mmol/l (Fig. 7d–f). In depth analysis of EGF action (ESM Table 2) revealed a mainly (approximately 2/3 of the effect) switch-like (digital) activation (i.e. activating inactive cells) and to a minor extent (approximately 1/3 of the effect) a graded (analogue) response (i.e. enhancing the activity of activated cells). Glucose elicited a mainly graded response (ESM Fig. 8) and enhanced the maximum EGF response (Fig. 7g–o). NEFA or increases in extracellular osmolality exerted no effect (Fig. 7p–x). Simultaneous exposure to high glucose and oleate exerted a similar effect as glucose alone (ESM Fig. 8).

Finally, we repeated all experiments performed with HK-2 cells using HEK cells and obtained virtually the same results (ESM Table 2, ESM Fig. 8b and c).

**Fig. 8:**
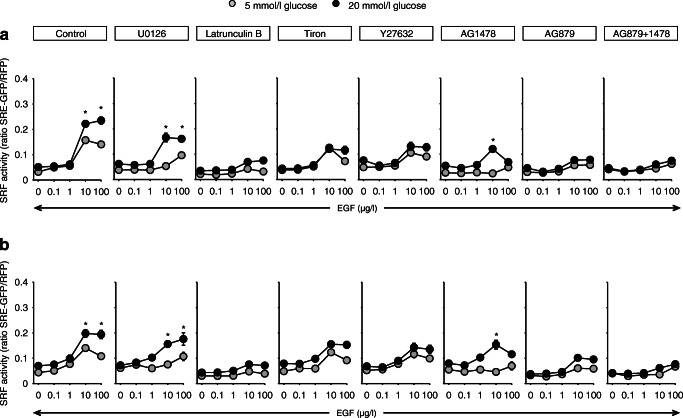
(**a**) Pathways involved in the glucose effect on EGF-induced SRF activation in HK-2 cells. (**b**) Pathways involved in the glucose effect on EGF-induced SRF activation in HEK cells. (**a**, **b**) Inhibition of ERK1/2 by 1 μmol/l U0126, inhibition of the actin-MRFT-pathway by 1 μmol/l latrunculin B, scavenging of ROS by 1 mmol/l tiron, inhibition of ROCK by 10 μmol/l Y27632, inhibition of EGFR with 100 nmol/l AG1478, inhibition of ErbB2 with 100 nmol/l AG879. Numbers for experiments: control HEK *n* = 24; AG879 + AG1478 *n* = 12; all other groups *n* = 18. *p < 0.05 vs 5 mmol/l glucose under otherwise the same conditions. *x*-axes are plotted on a log scale

In HK-2 and HEK cells, inhibition of ERK1/2 activation by U0126 reduced EGFR signalling substantially under normal glucose conditions at EGF concentrations up to 10 μg/l (Fig. 8a,b; comparison with the values in the presence of U0126 with those under control condition for 5 mmol/l glucose). In the presence of high glucose, EGF responsiveness of the cells in the presence of U0126 was restored (Fig. 8a,b; comparison of the values under low and high glucose conditions in the presence of U0126), i.e. high glucose rendered the cells ERK1/2-independent with respect to EGFR-mediated activation of SRF. High glucose concentrations exerted no effect on EGF-induced ERK1/2-phosphorylation (ESM Fig. 9).

Inhibition of signalling via MRTF by latrunculin B (Fig. 8a,b) reduced EGFR-induced SRF activation under normal and high glucose conditions (comparison with the respective values under control condition and of the values within the latrunculin group). The ROS-scavenger tiron mitigated EGF potentiation by glucose (Fig. 8a,b; comparison of the values within the tiron group). Inhibition of EGFR by AG1478 abolished the effect of up to 10 μg/l EGF under normal glucose conditions (Fig. 8a,b; comparison to the control group). Under high glucose conditions EGF was still active in the presence of AG1478 (Fig. 8a,b; comparison of the values within the AG1478 group), although to a lower degree (Fig. 8a,b). Inhibition of ErbB2 by AG879 reduced the effect of EGF under normal glucose as well as the synergistic effect of high glucose (Fig. 8a,b; comparison with the respective values under control condition and comparison of the values within the AG879 group), indicating the involvement of EGFR–ErbB2 heterodimers. The fact that EGF leads to the downregulation of EGFR and ErbB2 under high glucose (ESM Fig. 9) supports this conclusion. High glucose enhanced the expression of ErbB2 in HK-2 and HEK cells (ESM Fig. 9). Finally, inhibition of the Rho–ROCK pathway by Y27632 (Fig. 8a,b; comparison with the respective values under control condition and comparison of the values within the Y27632 group) prevented SRF activation. Thus, the Rho–ROCK–actin–MRTF pathway seems to mediate the glucose–EGF synergism.

#### Glucose enhances EGF-induced transcriptional activity of EGR transcription factors

EGR proteins function as transcription factors and are canonical targets of SRF. EGF enhances the expression of EGR-1 and stimulates EGR transcriptional activity in a glucose-dependent manner in HK-2 and HEK cells (ESM Fig. 10a-b). By contrast to SRF and EGR, we observed no effect on activator protein 1 (AP1)- or NFκB-reporter activity (ESM Fig. 11), excluding non-specific effects on the reporter.

## Discussion

From our results, which concur with pharmacological findings [50–54], we conclude that VSM-EGFR is involved in vascular dysfunction and nephropathy during HFD/type 2 diabetes. HFD-induced endothelial dysfunction and vascular transcriptome alterations depend on VSM-EGFR. Furthermore, VSM-EGFR plays a substantial role during HFD-induced renovascular alterations.

In WT animals, HFD induced the expected vascular phenotype [55–57], i.e. endothelial dysfunction and reduced responsiveness to phenylephrine [58]. The changes of the mRNA transcriptome correspond to vascular wall remodelling (via the Wnt–activator protein 2 [AP2] pathway) and to disturbed energy metabolism due to mitochondrial dysfunction. Thus, the gene sets that overlap significantly with identified genes are related to disturbed extracellular matrix homeostasis, leading to vascular stiffness and finally wall hypertrophy. Mitochondrial dysfunction is known to result in disturbed redox homeostasis, increased ROS formation and thereby vascular dysfunction, most prominently endothelial dysfunction.

The importance of EGFR for mitochondrial function and dysfunction was confirmed in cultured cells, showing that: (1) *Egfr*-KO reduces maximal respiration; (2) EGF reduced glycolysis rate; and (3) EGF and EGF+glucose stimulate respiration. In addition EGFR is involved in oleate-induced mitochondrial dysfunction. These alterations are most probably responsible for deranged homeostasis in the vascular wall and finally for the observed endothelial dysfunction. Deletion of VSM-*Egfr* mitigated HFD-induced vascular functional, structural and transcriptome alterations drastically, reducing the number of affected mRNAs to <10%. Consequently, there was no enrichment in GO terms or TFBS, nor in IPA pathways. Functionally, this corresponds well to the protection of vessels in VSM-*Egfr*-KO and to the alterations of mitochondrial function at the cellular level. Our data strongly suggest VSM-EGFR as prerequisite for HFD-induced transcriptome alterations followed by vascular remodelling.

Diabetic nephropathy is a leading cause of chronic renal failure [59], characterised by the accumulation of extracellular matrix in the glomerular and tubulointerstitial compartments and by remodelling of intrarenal vasculature, probably involving all renal cell types. Endothelial dysfunction and tubulointerstitial fibrosis have been proposed as key mechanism [60]. In line with the literature, HFD induced albuminuria and impaired glomerular filtration in WT but not KO animals. These data are in line with a pathogenic relevant role of VSM-EGFR for renal alterations during obesity. Although HFD-induced interstitial fibrosis or glomerular sclerosis were not prevented, media thickening in arterioles was absent in KO, suggesting that VSM-EGFR contributes to renovascular but not interstitial alterations.

We propose that the protective effect of VSM-*Egfr*-KO on vascular remodelling leads to partial prevention of renal damage, due to normalisation of glomerular haemodynamics and function. Furthermore, we suggest that vascular effects of HFD favour the development of tubule interstitial inflammation, which is reduced in KO animals. The renoprotective effect of VSM-*Egfr*-KO comprises the development of albuminuria, a trait resulting from changes of endothelial and filtration barrier function [61]. These results correspond to our observation that VSM-*Egfr*-KO prevented HFD-induced endothelial dysfunction.

HFD induced an inflammatory renal phenotype in WT mice, as already suggested before [59], which was prevented in KO animals. Thus, the significantly enriched gene sets in WT animals relate well to renal pathophysiology. The chronic inflammatory response in the tubule interstitial space is a strong trigger for fibroblast activation and epithelial mesenchymal transition, both leading to fibrotic alterations. The renal transcriptome of KO mice was affected in a qualitatively different manner, resulting in the upregulation of proximal tubular transport processes, especially for carboxylates. Upregulation of proximal tubular transport is known to be accompanied by cell enlargement or substrate accumulation (appearing as vacuolisation) and can explain the increased kidney size of KO animals fed an HFD [62, 63]. The mechanisms and/or triggers leading to a more pronounced upregulation of the renal transport transcriptome in KO animals are currently unknown. One possibility is an enhanced tubular substrate load due to preserved glomerular filtration. The relevance of this potential compensatory hypertrophy has to be investigated in future studies. Furthermore, it will be important to investigate the renal inflammatory phenotype in more detail, because it is known that HFD enhances the infiltration of inflammatory cells [64] and that macrophages act as key players in inflammation and fibrosis [65].

At the cellular level, high glucose but not enhanced levels of NEFA potentiate SRF-mediated transcriptional actions of EGFR. These findings concur with data from the literature that describe SRF as a switching substation for glucose-induced alterations of gene expression, linked to insulin resistance and obesity [25, 66, 67]. Such a role for SRF is supported by our transcriptome pathway analysis, which identified SRF as an activated vascular transcription regulator during HFD in WT animals. Thus, SRF integrates signalling from EGFR and hyperglycaemia in VSMCs with potential pathological relevance. Because NEFA do not enhance SRF activity we assume that SRF is not involved in oleate-induced mitochondrial dysfunction which, in turn, is not responsible for SRF activation. By contrast, EGF and EGF+high glucose activate SRF and enhance mitochondrial function. Because SRF is known to influence mitochondrial shape, motility and function [24], it is conceivable that it also mediates the effect of EGF and glucose on mitochondrial function. However, we cannot provide a definitive proof for this hypothesis. EGFR signalling and SRF activation are redox-sensitive [68] offering an explanation for the potentiating effect of hyperglycaemia. As enhanced mitochondrial function is accompanied by enhanced formation of ROS, a vicious cycle would arise. Future studies will need to test these hypotheses in depth.

Glucose leads to a qualitative switch in EGFR-to-SRF signalling, focusing the information transfer on the ROCK–actin–MRTF pathway and involving the generation of ROS. Because the ability of glucose to induce ROS formation and its contribution to pathological renovascular alterations is well investigated [59, 69–72], we propose a mechanism by which glucose potentiates pathological EGFR-to-SRF signalling via ROS. Although the source(s) of ROS under these conditions were not explicitly determined, the data on proton leakage and MitoSox fluorescence indicate a mitochondrial contribution. Of course, this does not exclude the involvement of additional mechanisms, such as NADPH oxidase induction by advanced glycation end-products or impaired antioxidant mechanisms.

We suggest that hyperglycaemia enhances EGF signalling via EGFR–ErbB2 heterodimers, which seem to be the starting point of the pathological signalling cascade. Possibly, this synergism results in altered tissue homeostasis and functional as well as structural remodelling. In the absence of VSM-EGFR these events are prevented or attenuated and vascular remodelling is dampened, mitigating renal end-organ damage. Preservation of endothelial function or glomerular filtration barrier during HFD are examples shown in our study.

In summary, our results show that VSM-EGFR is required for comprehensive HFD-induced functional vascular remodelling, endothelial dysfunction and renal end-organ damage. Mechanistically, elevated glucose potentiates EGFR signalling to the nucleus via the EGFR/ErbB2–ROCK–actin–SRF pathway (ESM Fig. 12), thereby leading to alterations of the transcriptome, mitochondrial respiration and finally to vascular dysfunction. Activation of the ROCK–actin–MRTF-pathway is known to affect smooth muscle cell differentiation. However, we did not perform an in-depth analysis of alterations in vascular marker protein expression in vivo. The detailed pattern of differentiation regulation under our experimental conditions will need to be assessed in future studies.

## Electronic supplementary material

ESM (PDF 1.44 mb)

## Data Availability

The datasets generated during and/or analysed during the current study are available in: (1) share_it: the data repository of the academic libraries of Saxony-Anhalt. (10.25673/32049.2). And: (2) The Gene Expression Omnibus database with the study identity GSE144838. (https://www.ncbi.nlm.nih.gov/geo/query/acc.cgi?acc=GSE144838).

## Abbreviations

EGFR: EGF receptor (also known as ErbB1)
EGR: Early growth response
ErbB2: EGF receptor 2
ERK: Extracellular signal-regulated kinase
GO: Gene ontology
GSEA: Gene set enrichment analysis
HEK: Human embryonic kidney
HFD: High-fat diet
HRP: Horseradish peroxidase
IPA: Ingenuity Pathway Analysis
KO: Knockout
MAPK: Mitogen-activated protein kinase
MRTF: Myocardin-related transcription factor
OCR: Oxygen consumption rate
PAS: Periodic acid–Schiff’s reagent
RFP: Red fluorescent protein
ROCK: Rho-associated protein kinase
ROS: Reactive oxygen species
SFD: Standard-fat diet
SNAP: *S*-Nitroso-*N*-acetyl-dl-penicillamine
SRE: Serum response element
SRF: Serum response factor
TBS: Tris-buffered saline
TFBS: Transcription factor binding site
TFBSA: TFBS analysis
VSM: Vascular smooth muscle
VSMC: Vascular smooth muscle cell
WT: Wild-type
