# Supplementary material for: Knockout of vascular smooth muscle EGF receptor in a mouse model prevents obesity-induced vascular dysfunction and renal damage in vivo
Source: Diabetologia. 2020 Jun 17;63(10):2218–34. doi: 10.1007/s00125-020-05187-4 (PMC7476975; doi:10.1007/s00125-020-05187-4)

Supplementary Tables

ESM table 1

Sequences of primer used for quantitative RT-PCR.

gene	forward	reverse
18S	GTAACCCGTTGAACCCCATT	CCATCCAATCGGTAGTAGCG
Adam17	AGAGAGCCATCTGAAGAGTTTGT	CCCCTCCATGACTGTTTGCT
Adra1a	CAGGGCCTCCGCAGC	AGTGACTCTCAACTTGGCCG
Adra1b	GCCAAAACCTTGGGCATTGT	AAGTAGCCCAGCCAGAACAC
Agtr1a	CGCTTCGGCCAGCGTCAGTT	GCCAAGCCAGCCATCAGCCA
Col1a1	ACATGTTCAGCTTTGTGGACC	TAGGCCATTGTGTATGCAGC
Col3a1	TGGTAGAAAGGACACAGAGGC	TCCAACTTCACCCTTAGCACC
Col4a1	ACAAAAGGGTGATGCTGGAG	CTCCCTTTGTACCGTTGCAT
Ptgs1	GCATTGCACATCCATCCACTC	GCTGAGTTGTAGGTCGGAGG
Ptgs2	TTCCTCCCGTAGCAGATGAC	CAGGGATGTGAGGAGGGTAGA
Ccn2	GCTTGGCGATTTTAGGTGTC	CAGACTGGAGAAGCAGAGCC
Eln	CCTACCAGGCAGCAATTACG	CCAGCCCCTGGATAATAGACTC
Nos3	GACCCTCACCGCTACAACAT	CTGGCCTTCTGCTCATTTTC
ErbB1 (Egfr)	GACCTTCACATCCTGCCAGT	GCATGGAGGTCAGTCCAGTT
Erbb2	GTTCCTCCTCGCCCTCCT	GGCAGGTAGGTAAGCTCCAA
Erbb3	GTGCTGGGTTTCCTTCTCAG	TTACCCATGACCACCTCACA
Erbb4	AGGTAGTCATGGGCAACCTG	TCCCACGAATAATGCGTAAA
ETAR (Ednra)	TCCGAGGAGCTCTAAGGTGA	GTGGTGCCCAGAAAGTTGAT
ETBR (Ednrb)	TTTGCTCTCTGTTGGCTTCC	GCTTTGAACTTCAGGCAGGA
Fn1	TTAAGCTCACATGCCAGTGC	TCGTCATAGCACGTTGCTTC
Hbegf	GACCCATGCCTCAGGAAATA	TGAGAAGTCCCACGATGACA
Icam1	GCTACCTGCACTTTGCCCT	AAGGCTTCTCTGGGATGGAT
**MCP-1 (*CCL2*)**	AGGTCCCTGTCATGCTTCTG	TCTGGACCCATTCCTTCTTG
Mmp2	GTCGCCCCTAAAACAGACAA	GGTCTCGATGGTGTTCTGGT
Mmp9	GAAGGCAAACCCTGTGTGTT	AGAGTACTGCTTGCCCAGGA
Nox1	TCCATTTCCTTCCTGGAGTG	CCCAACCAGTACAGCCACTT
**Nox2 (*Cybb*)**	GCTGGGATCACAGGAATTGTC	CTTCCAAACTCTCCGCAGTCT
Nox4	CCTTGAACTGAATGCAGCAA	ACCACCTGAAACATGCAACA
**Osteopontin (*SPP1*)**	ATTTGCTTTTGCCTGTTTGG	TGGCTATAGGATCTGGGTGC
**PAI1 (*Serpine1*)**	GACACCCTCAGCATGTTCATC	AGGGTTGCACTAAACATGTCAG
**RANTES (*CCL5*)**	CCCTCACCATCATCCTCACT	TCCTTCGAGTGACAAACACG
Tgfa	GCGGCTGCAGTGGTGTCTCA	GAGGGCACGGCACCACTCAC
Tgfb1	TTTGGAGCCTGGACACACAG	TGGACAACTGCTCCACCTTG
**Tissue factor (*F3*)**	AGGATGTGACCTGGGCCTAT	GGCTGTCCAAGGTTTGTGTC
Tnf	CACACTCAGATCATCTTCTCAAAA	GTAGACAAGGTACAACCCATCG
Vcam1	CCGGCATATACGAGTGTGAA	ACCAAGGAAGATGCGCAGTA

ESM table 2

Endogenous EGFR is mainly a digital activator of SRE signaling in HK-2 cells, i.e. recruitment of additional SRE-positive cells (N=80).

Effect of 10 µg/l EGF	SRE-activity of SRE-positive cells (% of control)	fraction of SRE-positive cells (% of control)	ratio Δdigital/Δanalogue
6h	116±7	169±10	4.3
24h	150±9	252±16	3.0
48h	157±14	230±13	2.3

Endogenous EGFR is mainly a digital activator of SRE signaling in HEK cells, i.e. recruitment of additional SRE-positive cells (N=136).

Effect of 10 µg/l EGF	SRE-activity of SRE-positive cells (% of control)	fraction of SRE-positive cells (% of control)	ratio Δdigital/Δanalogue
6h	123±10	161±15	2.7
24h	146±10	243±13	3.1
48h	156±6	246±15	2.6

Supplementary Figures

ESM figure 1 | Vascular gene expression analysis of EGFR singalling members and the transcription factors SFR and SP1. | N = 4 animals per experimental group.

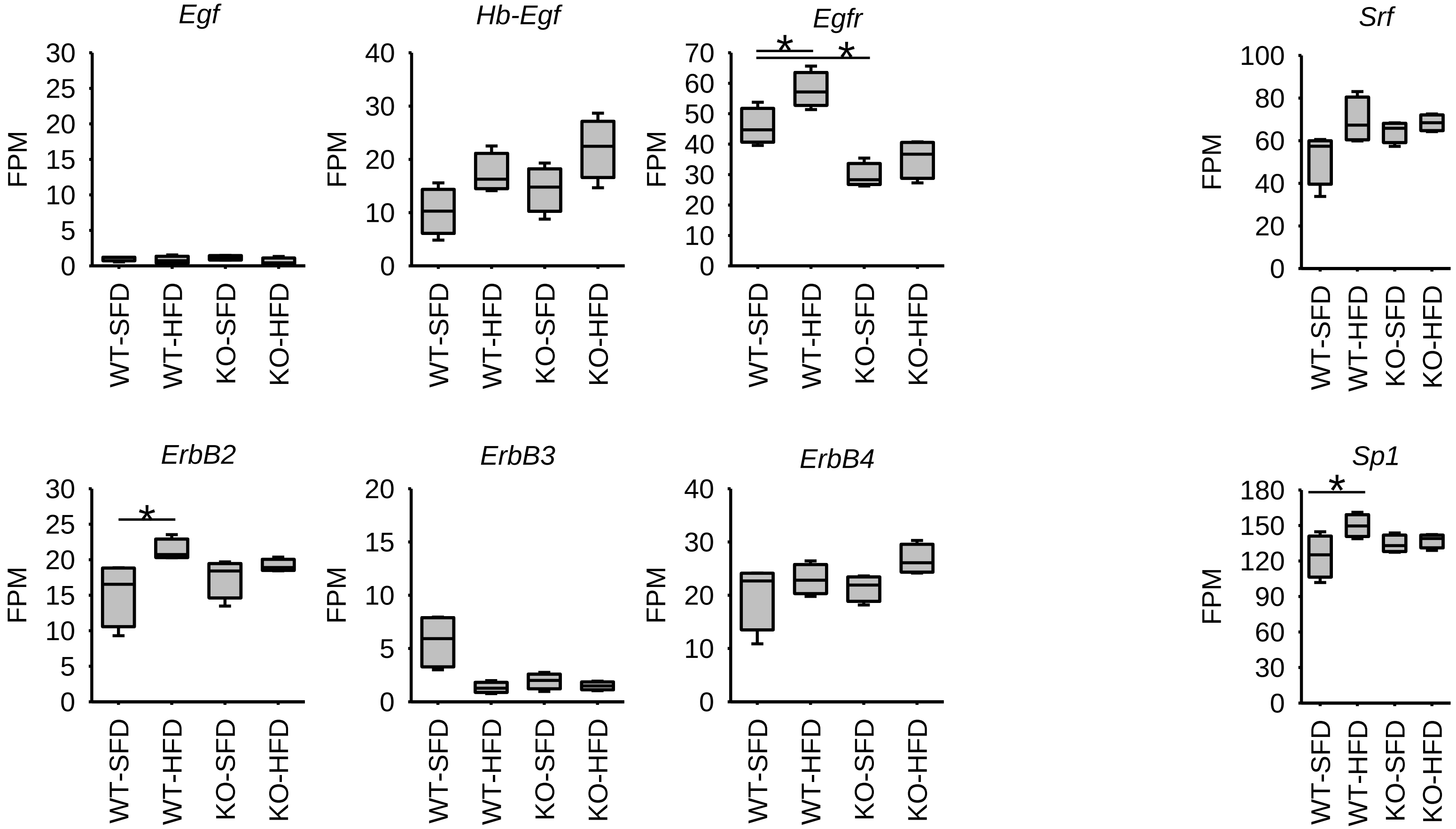

ESM figure 2 | Vascular gene expression analysis | Changes in the Wnt-pathway according to the RNA-seq results for upregulated genes and their possible contribution to changes in extracellular matrix homeostasis. N=4 for each experimental group.

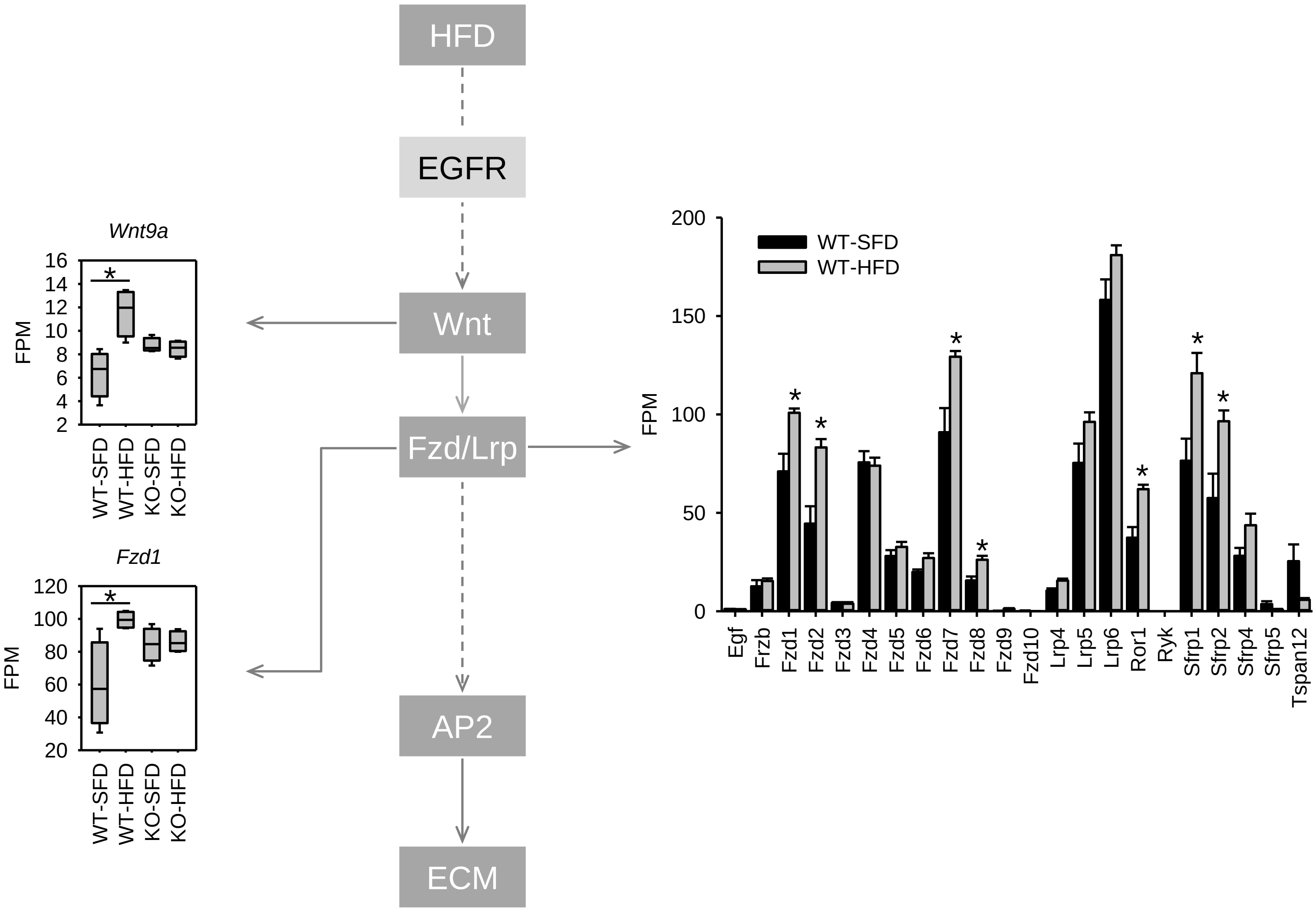

ESM figure 3 | Mitochondrial function of primary vascular smooth muscle cells isolated from aortae of WT or KO animals. Determined by the Seahorse® technology. N = 6.

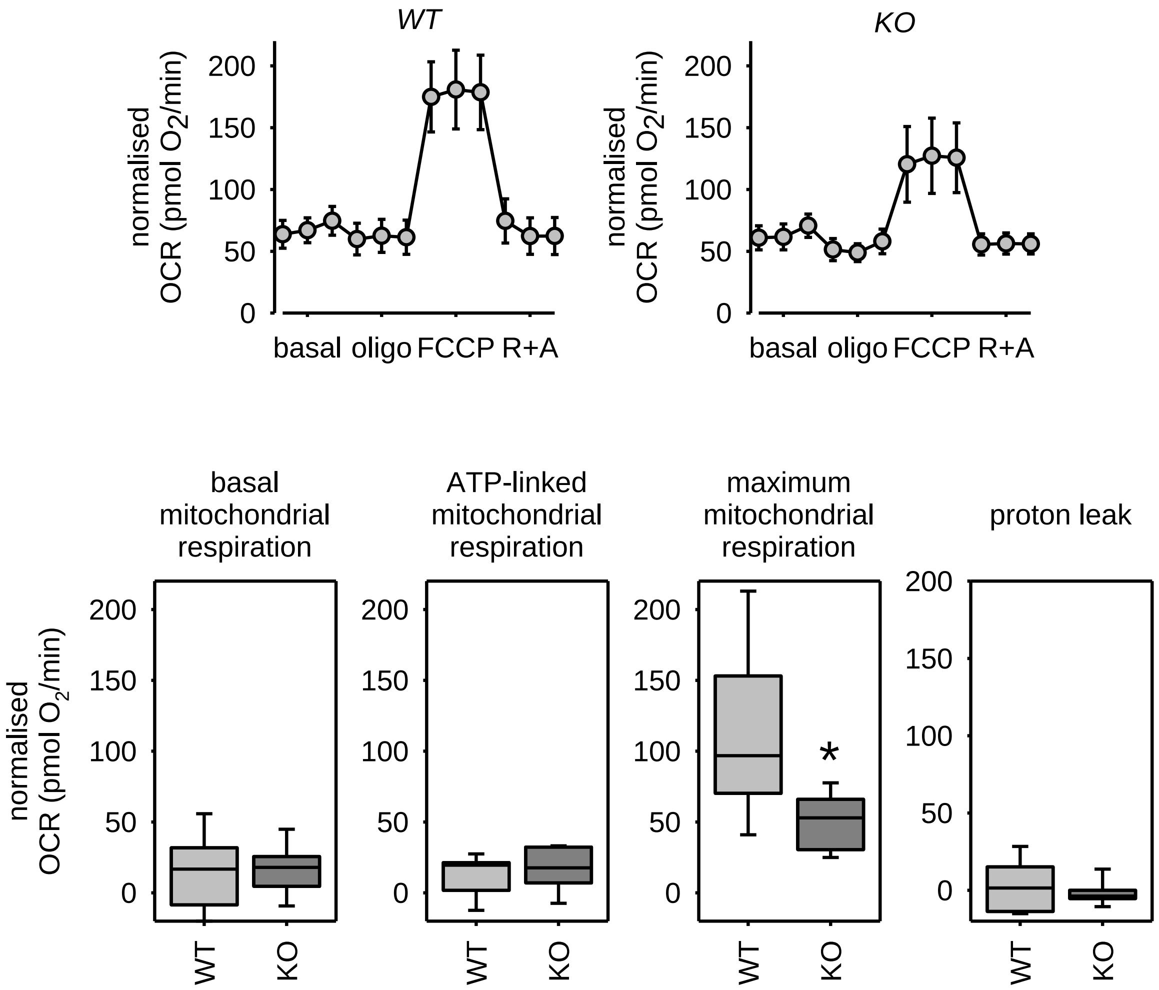

ESM figure 4 | Cardiac gene expression analysis. Number of animals: N = 10 for each experimental group. SD = standard diet; HFD = high fat diet.

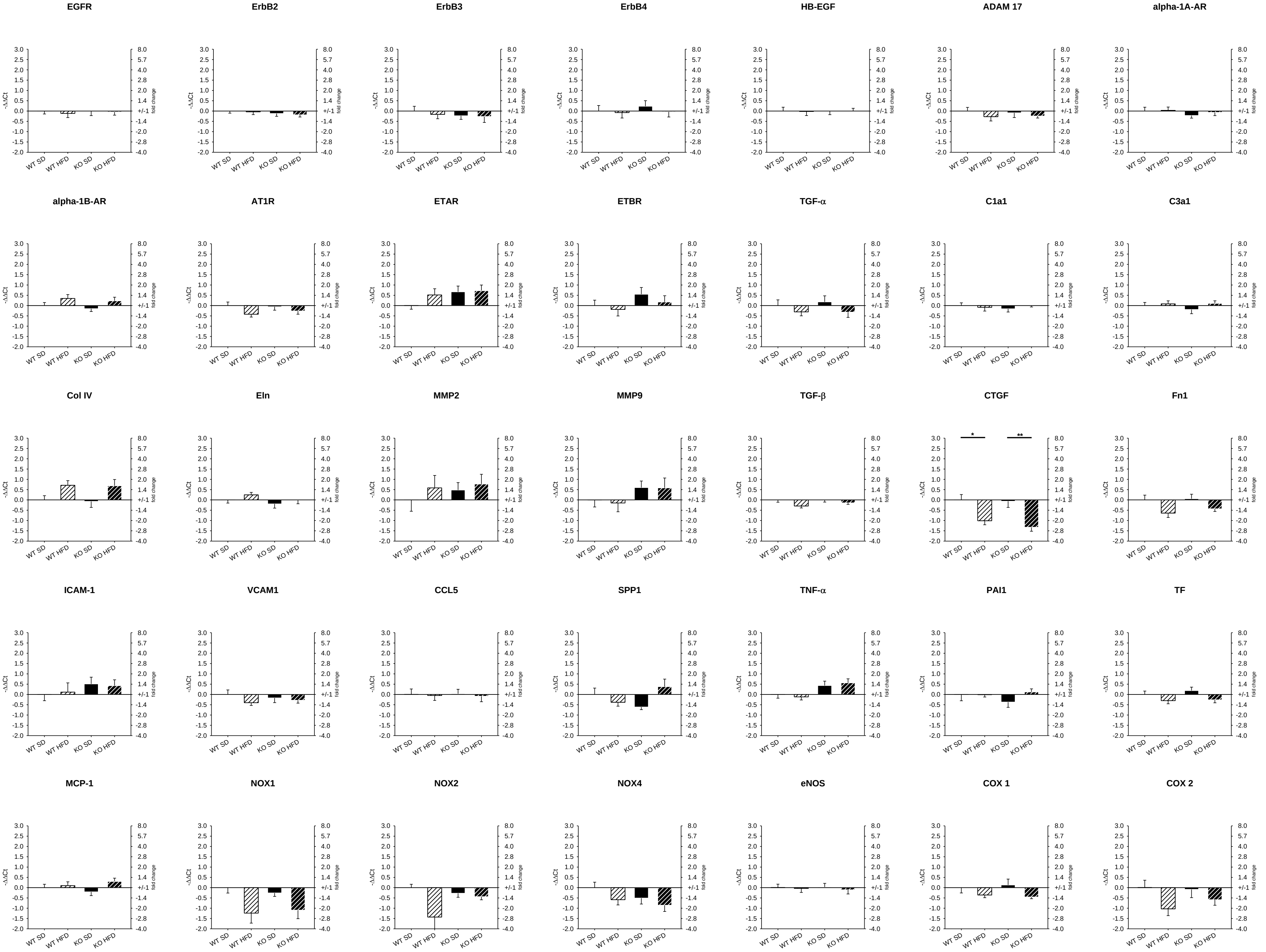

ESM figure 5 | Directed renal gene expression analysis. N = 10 for each experimental group. . * = p<0.05.

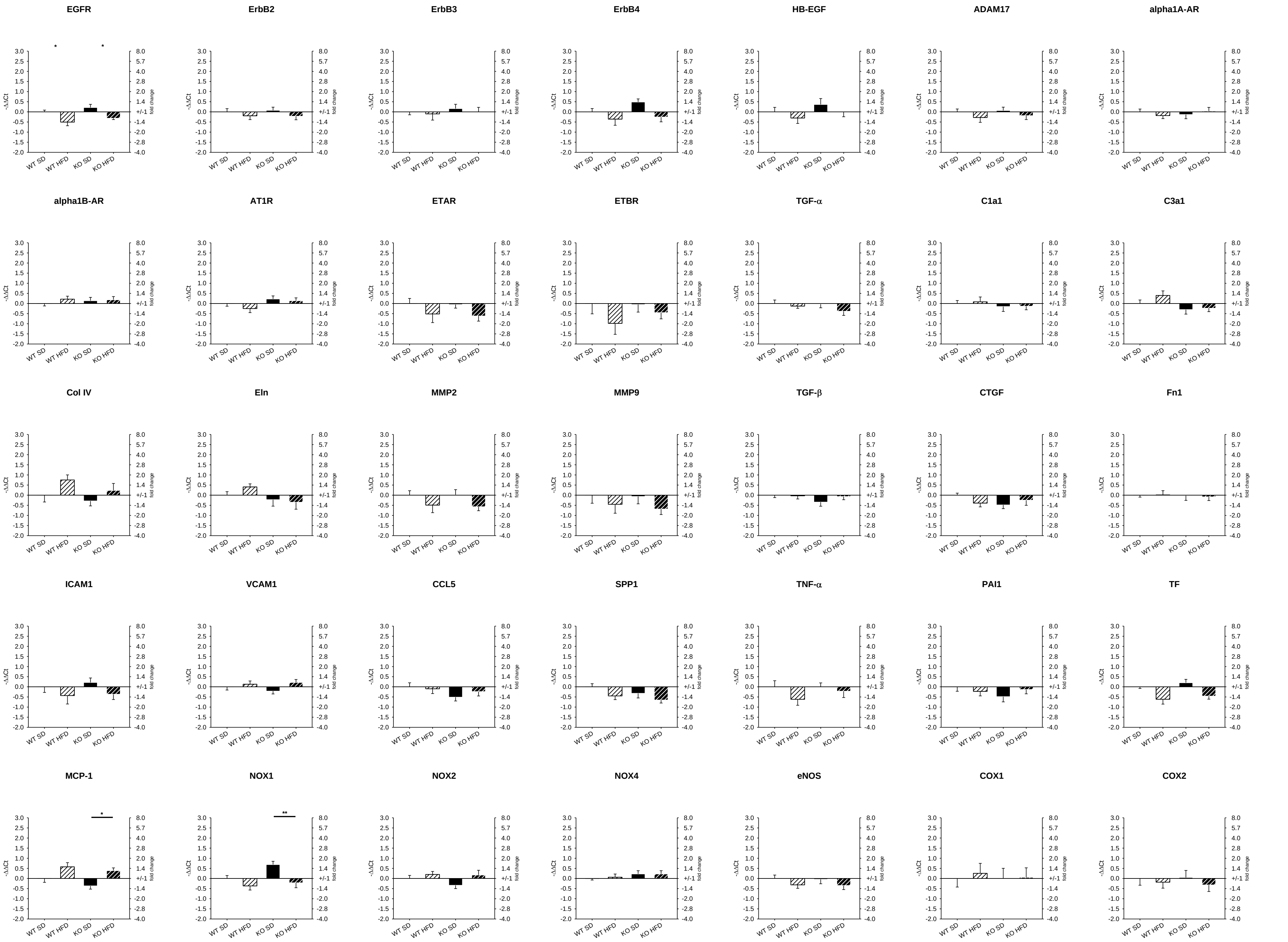

ESM figure 6 | Free fatty acids exert no synergistic action with respect to EGF-induced SRF activation (a) or ERK1/2 phosphorylation (b) in A7r5 cells. Furthermore, incubation with oleate under high glucose conditions did not result in SRF activation (c). * = p<0.05 versus control. 10 µg/l EGF, 100 µmol/l oleate.

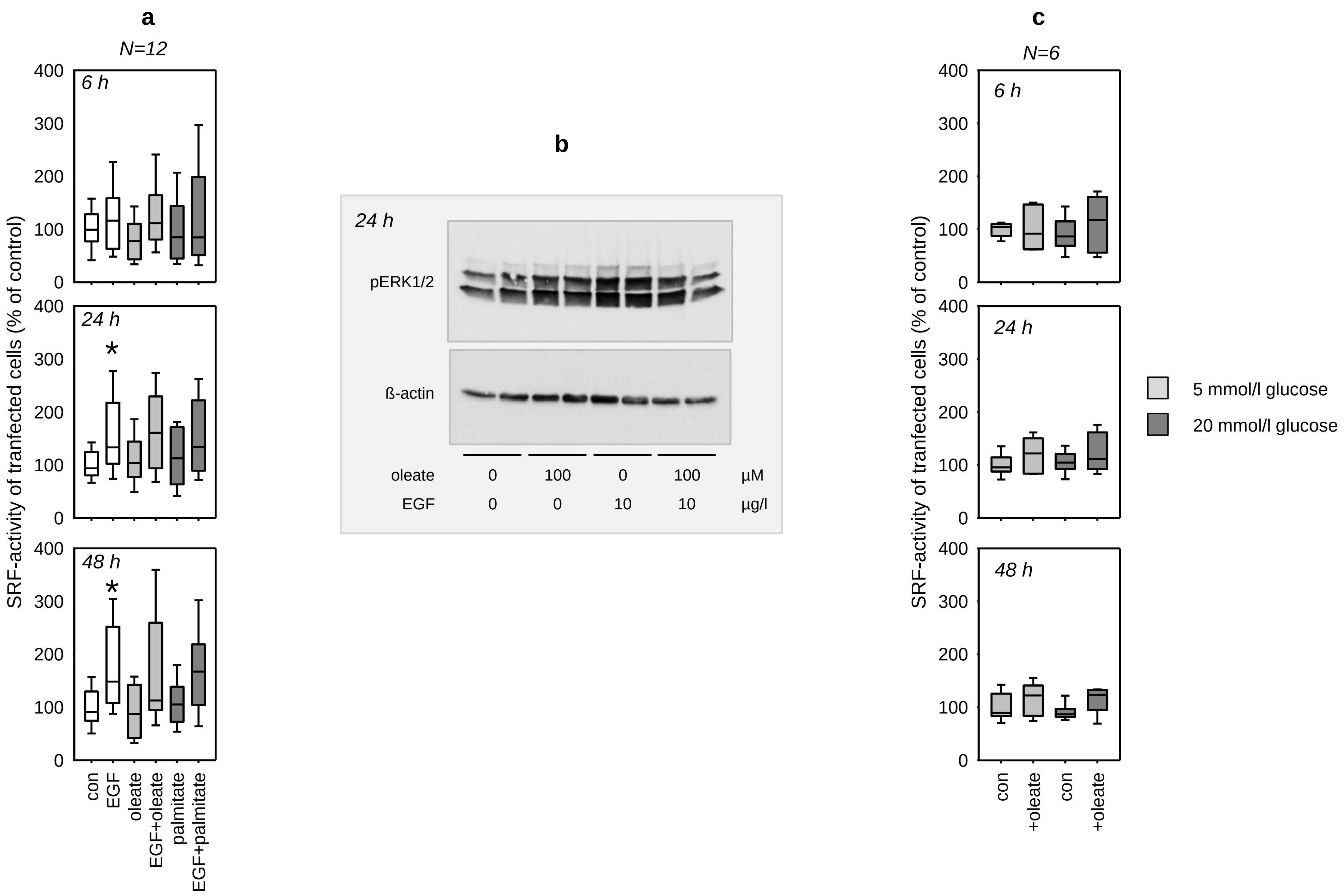

ESM figure 7a | Western blot analysis of EGFR, ErbB2, SRF, pERK1/2-expression in A7r5 cells. High glucose did not enhance the effect of EGF. N = 6. * = p<0.05.

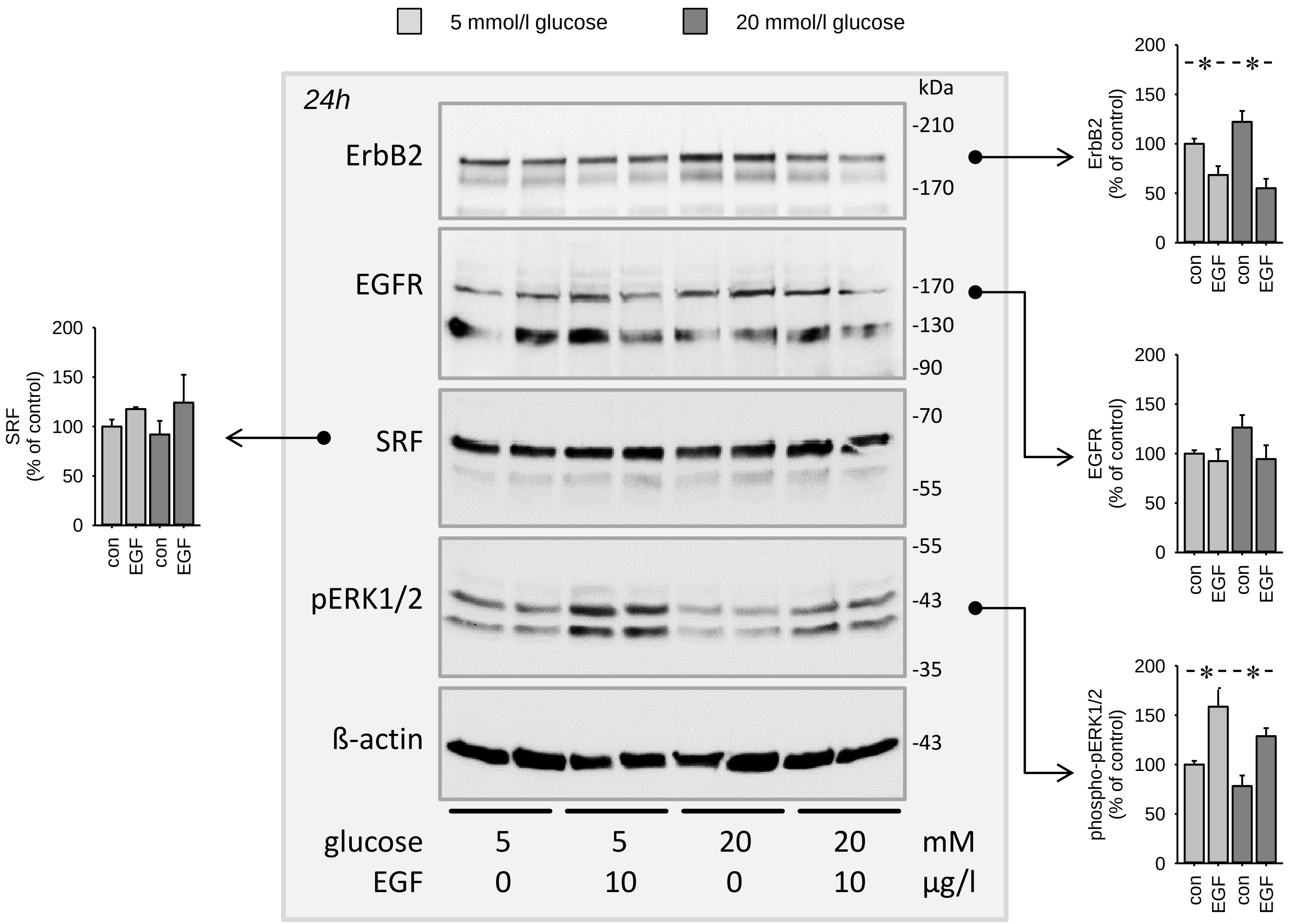

ESM figure 7b | Western blot analysis of EGFR, ErbB2, SRF, pERK1/2-expression in A7r5 cells. High glucose did not enhance the effect of EGF. N = 8. * = p<0.05.

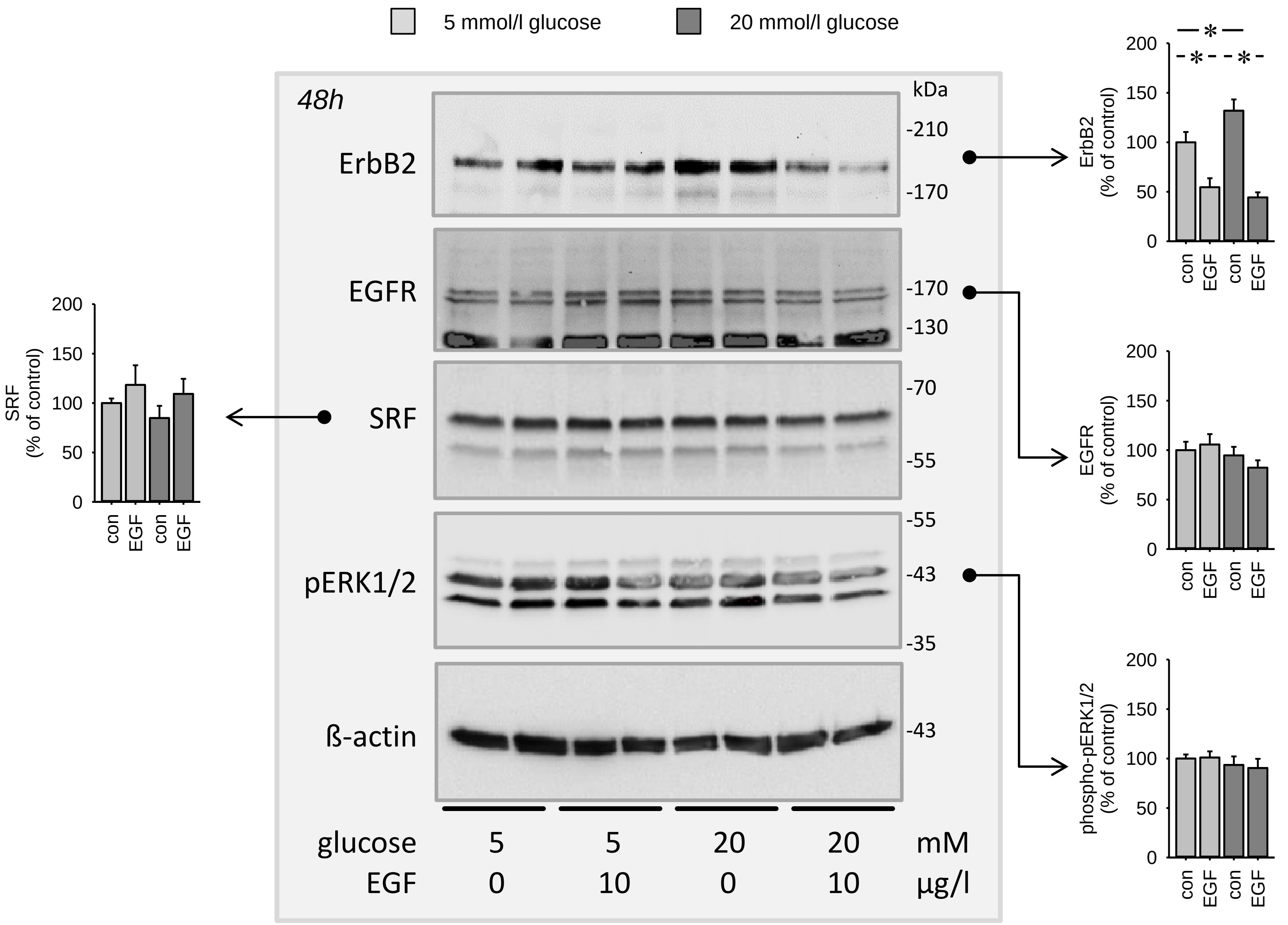

ESM figure 7c | Western blot analysis of protein expression and ERK1/2 phosphorylation in primary mouse aorta smooth muscle cells. High glucose (48 h exposure) did not affect EGFR expression or ERK1/2 phosphorylation induced by 1 µmol/l PMA or 10 µg/l EGF (5 min exposure). N = 4.

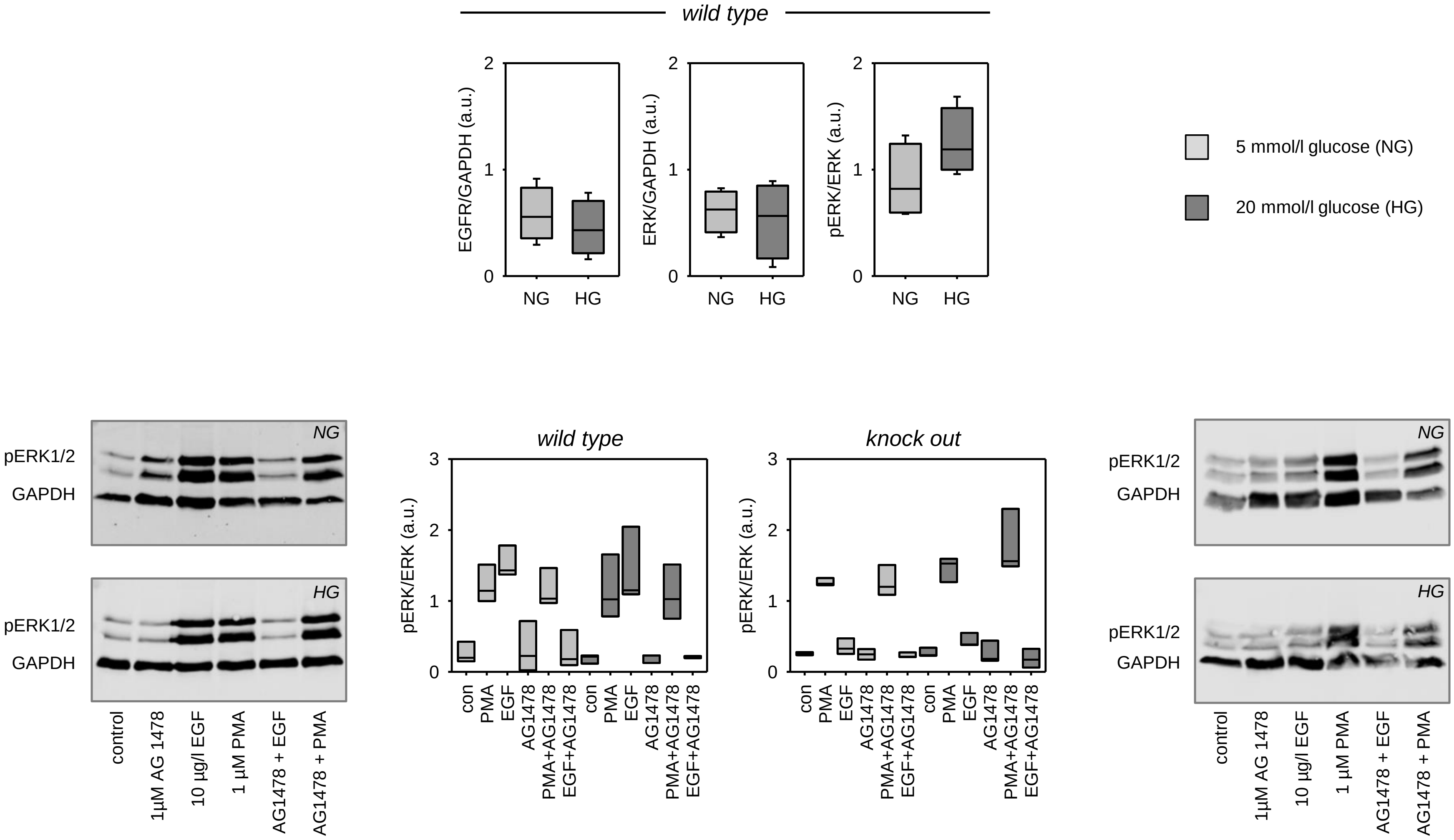

ESM figure 8a, b | Enhancement of EGF-induced SRE-activity by glucose in HK-2 (A) and HEK (B) cells. Glucose enhances SRE activity concentration-dependent in the presence of EGF. This is mainly an analogue effect, i.e. enhanced activity in cells activated by EGF. Glucose enhances EGF-induced SRF activation also in the presence of the free fatty acid oleate. N=12.

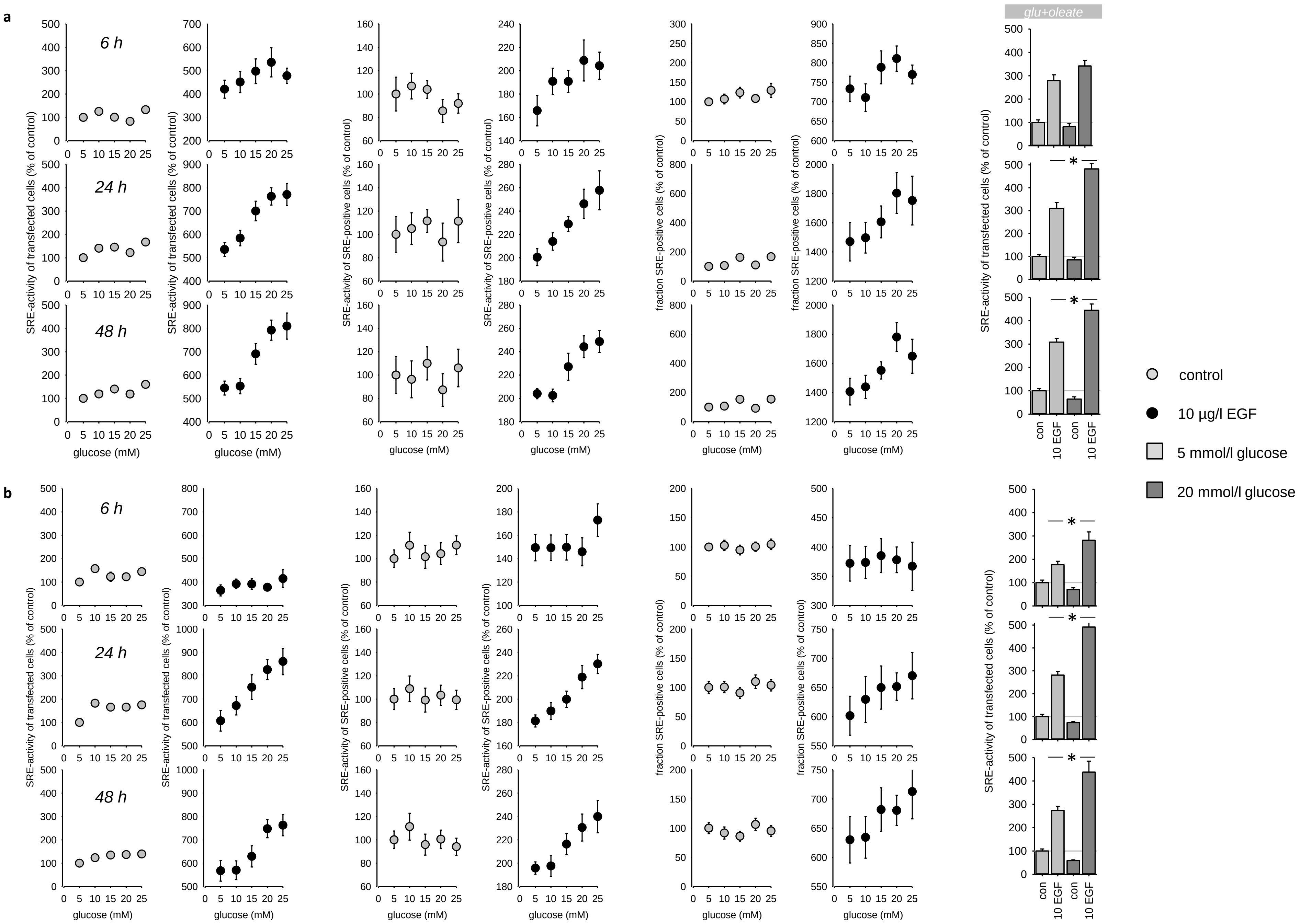

ESM figure 8c | i) Stimulation of SRF-activity in HEK cells by EGF and glucose. ii) In the presence of EGF, glucose enhances SRF-activity concentration dependent. iii-v) Dose-response curves of EGF-induced SRF-activity in HEK cells and its alteration by high glucose conditions. Glucose enhances SRF activity mainly by an analogue effect, i.e. enhanced activity in cells already activated by EGF. vi-viii) Oleate, palmitate or mannit exert no stimulatory effect on SRF-activity. Con = medium without serum. * = p<0.05 versus respective control if not indicated otherwise.

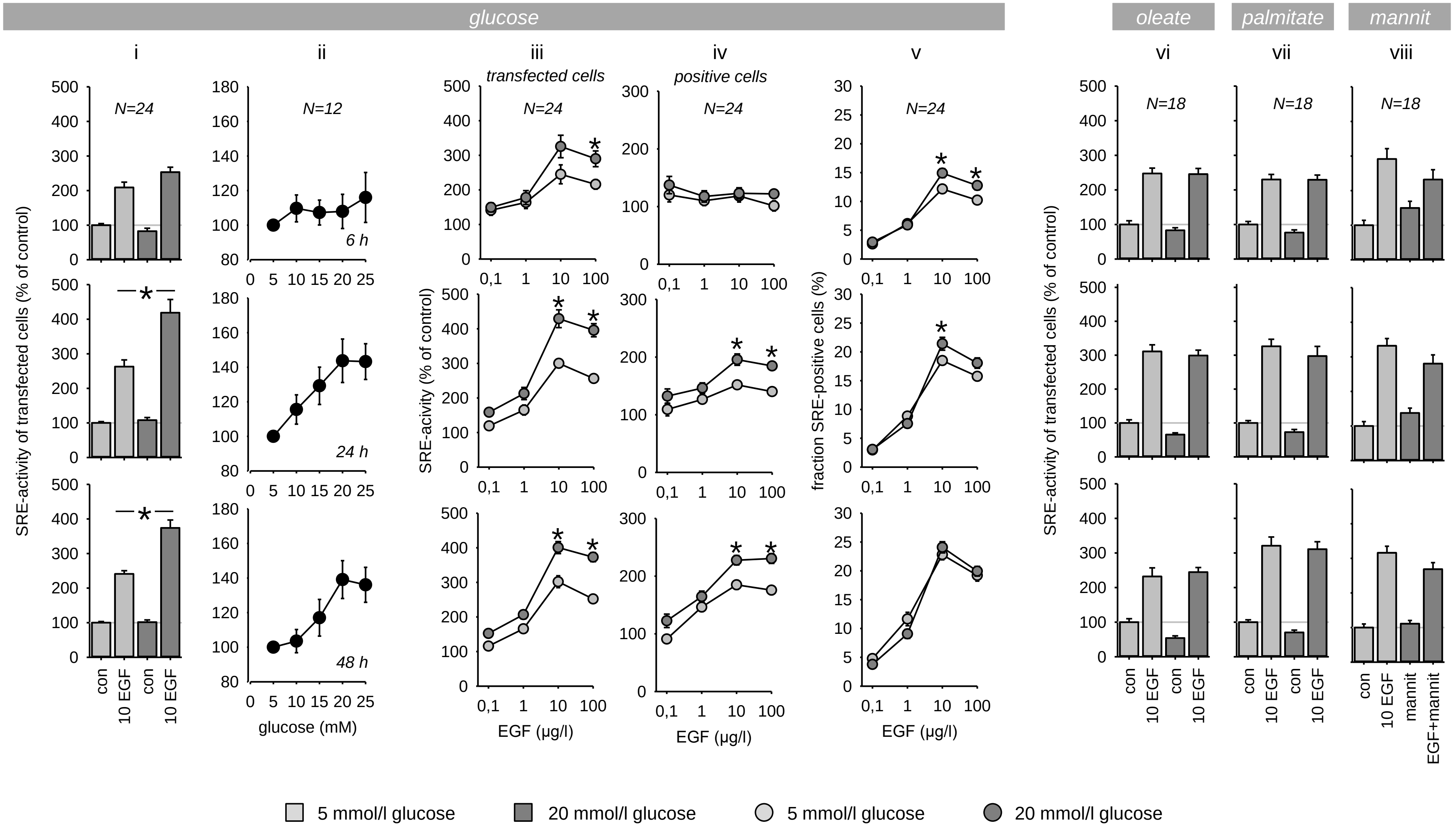

ESM figure 9 | Effect of 6h EGF and high glucose exposure on ERK1/2 phosphorylation, EGFR, ErbB2 and SRF expression in HK-2 and HEK cells. N=8.

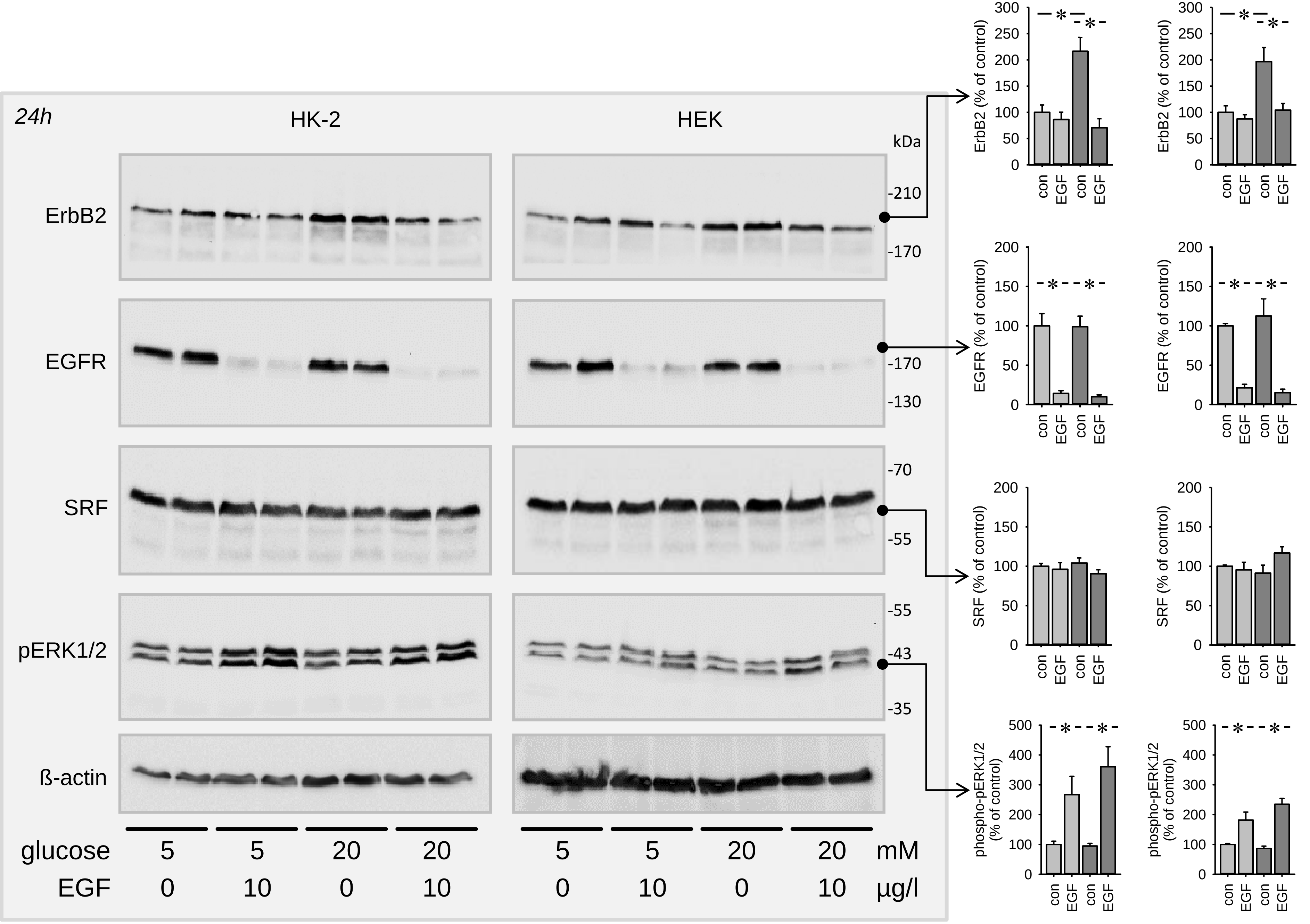

ESM figure 10a | Concentration-dependent stimulation of EGR-activity in HK-2 cells by glucose. In the presence of EGF glucose stimulates EGR-activity, mainly by enhancing the activity of EGR-postive cells (analogue signaling), and EGR-1 expression. ⁎ = p<0.05 versus control or 5 mmol/l glucose.

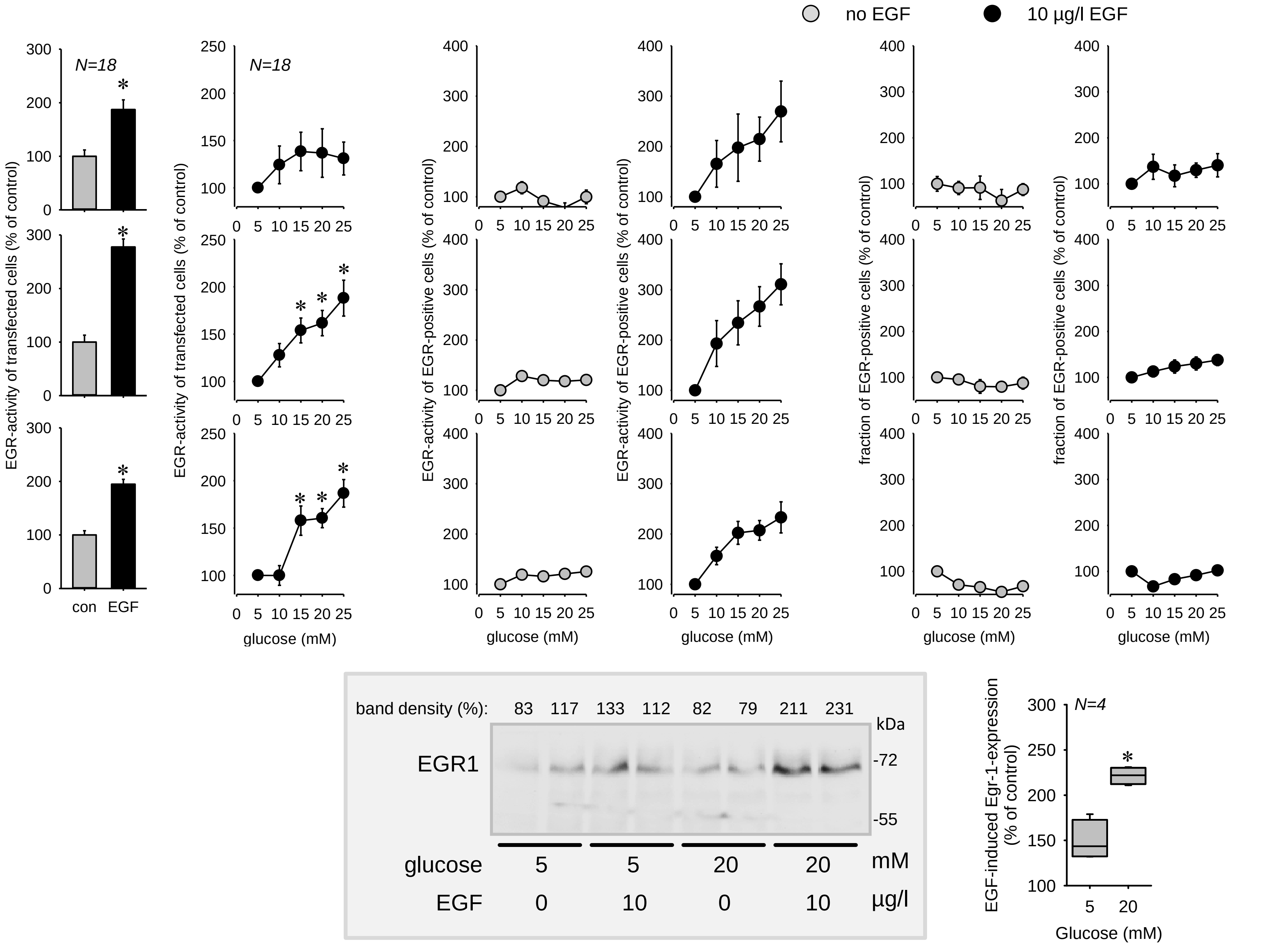

ESM figure 10b | Concentration-dependent stimulation of EGR-activity in HEK cells by glucose. In the presence of EGF glucose stimulates EGR-activity, mainly by enhancing the activity of EGR-positive cells (analogue signaling), and EGR-1 expression.. ∗ = p<0.05 versus control or 5 mmol/l glucose.

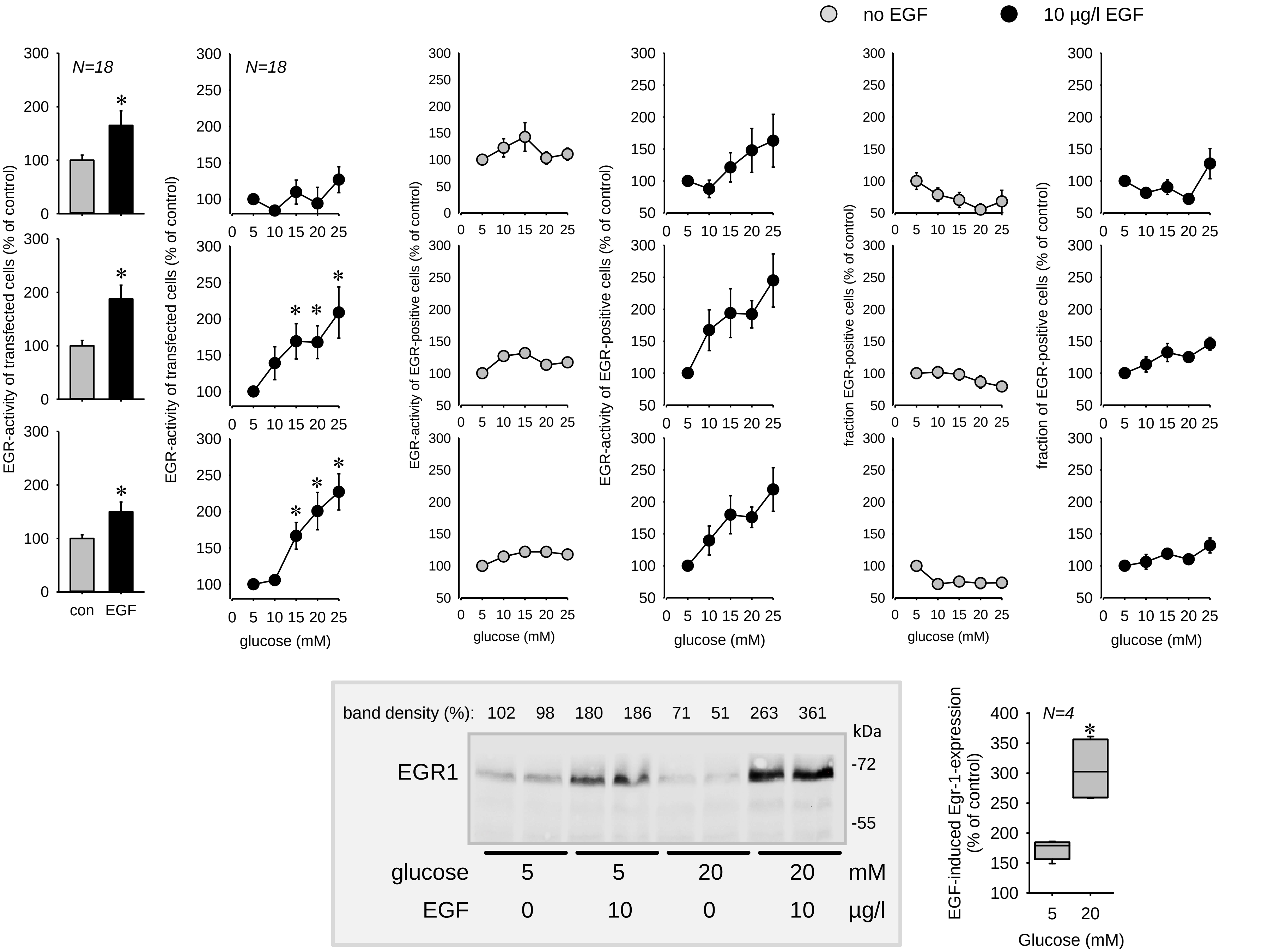

ESM figure 11 | No effect of EGF or glucose on AP1- or NFkB-activity could be detected in HK-2 and HEK cells.

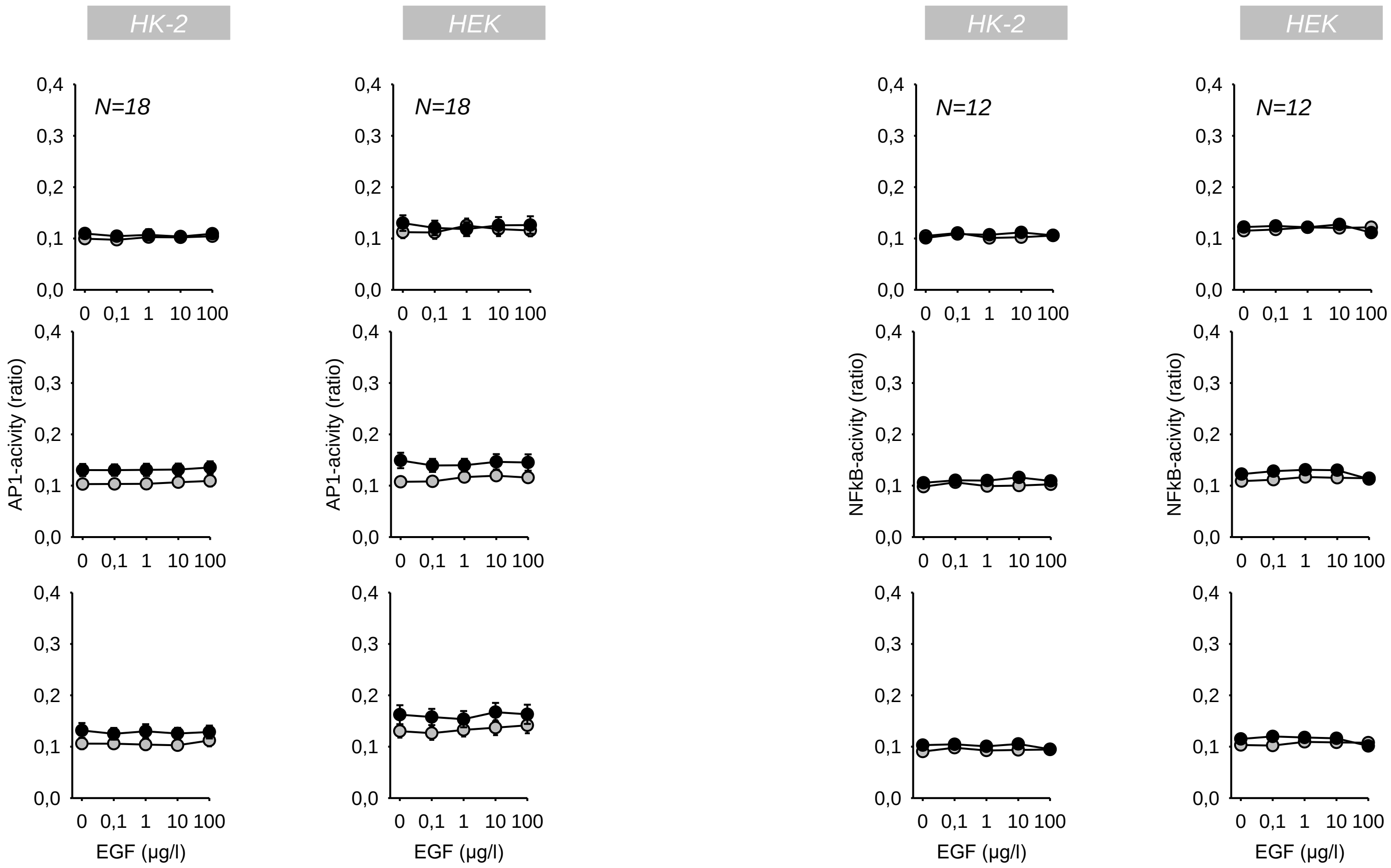

ESM figure 12 | Graphical abstract

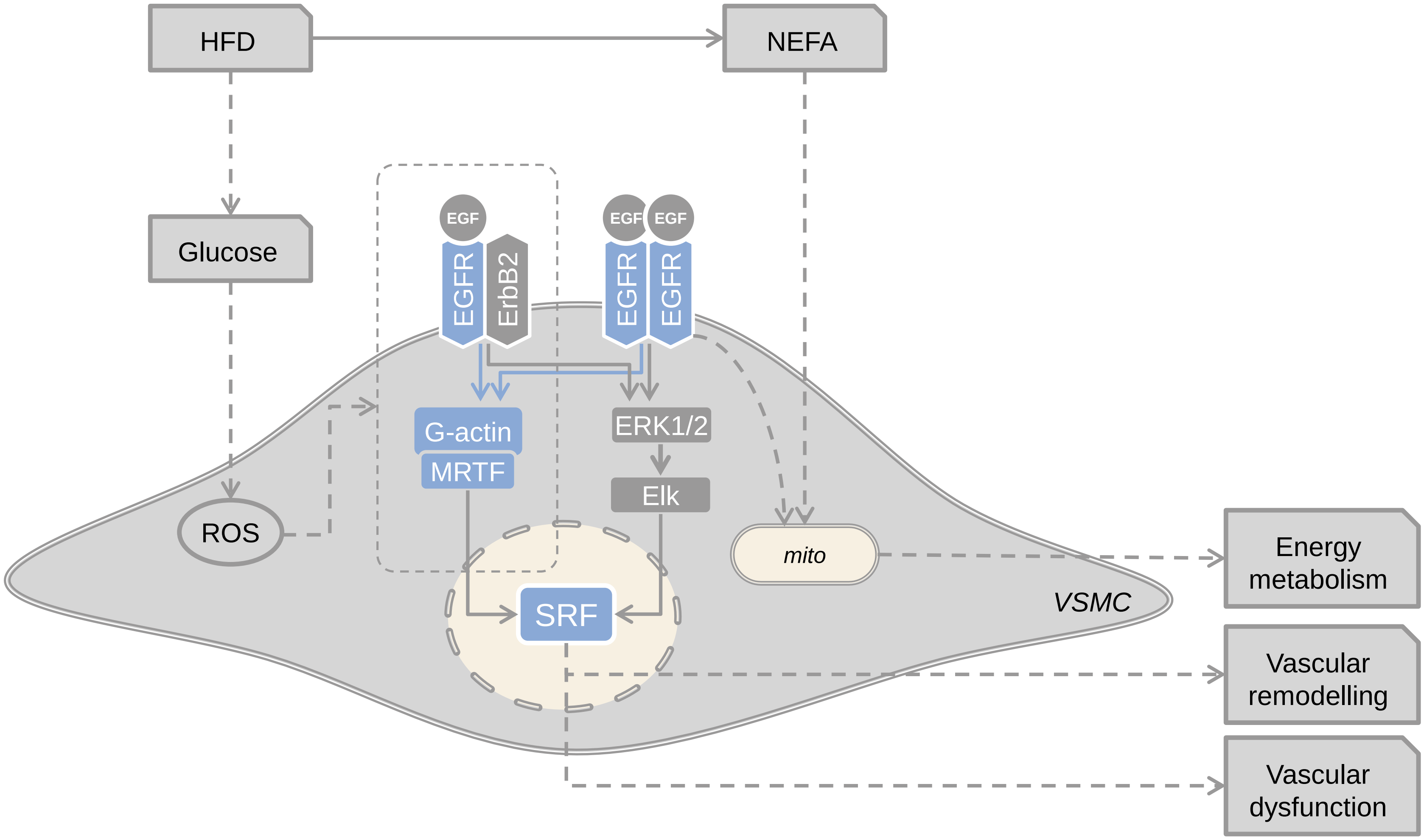

Supplement: Supplementary file 1 — (PDF 1.44 mb) [file 125_2020_5187_MOESM1_ESM.pdf]
